# Supplementary material for: Unified tumor growth mechanisms from multimodel inference and dataset integration
Source: PLoS Comput Biol. 2023 Jul 5;19(7):e1011215. doi: 10.1371/journal.pcbi.1011215 (PMC10351715; doi:10.1371/journal.pcbi.1011215)
Supplement: S1 Text — Note A. Contrasting AIC vs posterior probability calculated by Bayes-MMI for model selection and multi-model inferenceA.1. Using multiple models to evaluate how well a variable informs the observed data: an example. A.2. Marginal likelihood or “evidence” is calculated using model optimization followed by Bayes’ Theorem. A.3. AIC is calculated as an estimate of the Kullback-Liebler divergence. A.4. Notable differences between AIC and Bayesian evidence / posterior probability. A.5. Model selection allows us to evaluate which variables or terms have the largest effect on observed data. A.6. Model averaging uses model selection outcomes from all models to demonstrate how the observed data informed the model variables or terms that represent our hypotheses. A.7. Advantages of Bayes-MMI over AIC for model selection and model averaging: continuing example. Note B. Sums of AIC weights (SW) and posterior probability on a subset of the candidate models. Note C. Bayesian Information Criterion. Table A Summary of nested sampling model selection results on the simulated dataset and model selection problem in Galipaud et al., 2014, ranked by AICc. Table B Summary of nested sampling model selection results on the simulated dataset and model selection problem in Galipaud et al., 2014, ranked by posterior probability. Table C SW and posterior probability calculations for each model variable in both full candidate set and partial candidate set examples. Table D Summary of AICc and nested sampling model selection results using a partial candidate set. Table E Summary of nested sampling model selection results on the simulated dataset and model selection problem in Galipaud et al., 2014, ranked by BIC-estimated posterior probability. Table F Comparing BIC-estimated probability and marginal likelihood-calculated probability for each model variable in full and partial candidate sets. (DOCX) [file pcbi.1011215.s001.docx]

**S1 Text. Didactic example contrasting Akaike Information Criterion and Bayesian posterior probability**

**Note A.** **Contrasting AIC vs posterior probability calculated by Bayes-MMI for model selection and multi-model inference**

**A.1 Using multiple models to evaluate how well a variable informs the observed data: an example**

Mathematical models are a useful way to interrogate a biological system. Building a model can help the investigator hypothesize relationships within the system, and more specifically evaluate whether and how these relationships relate to observed phenomena. Each piece of a model represents a distinct hypothesis; thus the model as a whole represents multiple hypotheses about the system of interest.

For example, a farmer might wish to determine which aspects of his farm most affect his monthly income: milk from his cows, eggs from his hens, wool from his sheep, or pony rides offered to the public. He is interested in assessing how he should price the product or service based on how many animals he must keep his income steady. Additionally, if a provided product or service doesn’t impact his monthly income, perhaps he will stop providing it. This is a question with one outcome or response variable, the monthly income, and up to four predictor variables, one each for the animals that provide the products or services. We can write a linear equation that describes these interactions as shown below:

$income=p_{milk}*cows+p_{eggs}*hens+p_{wool}*sheep+p_{rides}*ponies+savings interest$ (1)

$y= \beta_{1}x_{1}+\beta_{2}x_{2}+\beta_{3}x_{3}+\beta_{4}x_{4}+\beta_{0}$ (2)

(Eq. 2) represents a linear regression model corresponding to the more intuitively written (Eq. 1), where the savings interest represents the “intercept”, or a baseline amount of money where the farm budget starts each month. However, (Eq. 2) is only one of many possible models that the farmer could use to determine how these variables come together to yield his income and perhaps improve on his earnings. A multi-model averaging approach would enable the farmer to explore model hypotheses to explore how much he should charge, *β_1-4_*, based on number of working animals that month, *x_1-4_*, and his monthly income, *y*, *and*, how much each of those animals *x_1-4_* contribute to the income *y*.

Besides using linear regression to determine pricing, the farmer can evaluate all potential models including few, some, or all farm animals to see which animals most directly affect his income. Perhaps the lower-earning products per month are not needed to maintain the income based on the numbers of animals providing each product. For example, if sheep’s wool and pony rides do not bring in as much income as milk or eggs, maybe they do not contribute meaningfully to the income, and the best model would look like this:

$income=price_{milk}*cows+price_{eggs}*hens+savings interest$ (3)

$y= \beta_{1}x_{1}+\beta_{2}x_{2}+\beta_{0}$ (4)

With all possible combinations of animals that the farmer might use to support his income, there are 16 different possible models that can help predict his monthly income, below:

| $\boldsymbol{y=}\boldsymbol{\beta}_{\boldsymbol{0}}$ | $\boldsymbol{y=}\boldsymbol{\beta}_{\boldsymbol{1}}\boldsymbol{x}_{\boldsymbol{1}}\boldsymbol{+}\boldsymbol{\beta}_{\boldsymbol{2}}\boldsymbol{x}_{\boldsymbol{2}}\boldsymbol{+}\boldsymbol{\beta}_{\boldsymbol{0}}$ |  |
| --- | --- | --- |
| $\boldsymbol{y=}\boldsymbol{\beta}_{\boldsymbol{1}}\boldsymbol{x}_{\boldsymbol{1}}\boldsymbol{+}\boldsymbol{\beta}_{\boldsymbol{0}}$ | $y= \beta_{1}x_{1}+\beta_{3}x_{3}+\beta_{0}$ | $y= \beta_{1}x_{1}+\beta_{2}x_{2}+\beta_{3}x_{3}+\beta_{0}$ |
| $\boldsymbol{y=}\boldsymbol{\beta}_{\boldsymbol{2}}\boldsymbol{x}_{\boldsymbol{2}}\boldsymbol{+}\boldsymbol{\beta}_{\boldsymbol{0}}$ | $y= \beta_{1}x_{1}+\beta_{4}x_{4}+\beta_{0}$ | $y= \beta_{1}x_{1}+\beta_{2}x_{2}+\beta_{4}x_{4}+\beta_{0}$ |
| $\boldsymbol{y=}\boldsymbol{\beta}_{\boldsymbol{3}}\boldsymbol{x}_{\boldsymbol{3}}\boldsymbol{+}\boldsymbol{\beta}_{\boldsymbol{0}}$ | $y= \beta_{2}x_{2}+\beta_{3}x_{3}+\beta_{0}$ | $y= \beta_{1}x_{1}+\beta_{3}x_{3}+\beta_{4}x_{4}+\beta_{0}$ |
| $\boldsymbol{y=}\boldsymbol{\beta}_{\boldsymbol{4}}\boldsymbol{x}_{\boldsymbol{4}}\boldsymbol{+}\boldsymbol{\beta}_{\boldsymbol{0}}$ | $y= \beta_{2}x_{2}+\beta_{4}x_{4}+\beta_{0}$ |  |
|  | $y= \beta_{3}x_{3}+\beta_{4}x_{4}+\beta_{0}$ |  |
| $\boldsymbol{y=}\boldsymbol{\beta}_{\boldsymbol{1}}\boldsymbol{x}_{\boldsymbol{1}}\boldsymbol{+}\boldsymbol{\beta}_{\boldsymbol{2}}\boldsymbol{x}_{\boldsymbol{2}}\boldsymbol{+}\boldsymbol{\beta}_{\boldsymbol{3}}\boldsymbol{x}_{\boldsymbol{3}}\boldsymbol{+}\boldsymbol{\beta}_{\boldsymbol{4}}\boldsymbol{x}_{\boldsymbol{4}}\boldsymbol{+}\boldsymbol{\beta}_{\boldsymbol{0}}$ |  |  |
|  |  |  |

The model selection process that the farmer can use involves evaluating how well each candidate model’s (from this superset of plausible models) simulations of *pricing * animal* compared to the observations of his income. This process is often performed for choosing one best-matching model, and whichever model is best is often considered to include only the important model variables (the most important animals from the perspective of the farmer’s income. However, we and others argue, one may use model averaging, where parameter values (prices) can be weighted by model probability and then combined into a distribution of likely values, and similarly variable (animal) likelihood can be weighted and summed across all models (1,2). Thus, this set of candidate models can provide support for or against pieces of models, and therefore support for or against hypotheses.

What is the best framework for which the farmer can perform model selection and model averaging? We argue that a Bayesian framework is optimal, compared in this example to the most common model selection/model averaging framework, the information theoretic approach of AIC (1,3). In sections **A.2**-**A.6** we provide the background information that motivate our claim that a Bayesian framework should be optimal. In section **A.7**, we provide our Bayesian analysis of a published linear regression example that investigated AIC model selection and model averaging to compare the two frameworks.

**A.2** **Marginal likelihood or “evidence” is calculated using model optimization followed by Bayes’ Theorem**

In a Bayesian statistics context, probability indicates the degree of belief or confidence in an event, or in a proposal that a statement may be true – that is, a hypothesis. This prior degree of belief (hypothesis) is then updated with new data, which can yield evidence that our prior belief must be modified. In this case, data can refer to measurements related to the hypothesis and is synthesized into a likelihood that the hypothesis is true. Prior knowledge combined with data leads to the posterior, updated, probability, defined using Bayes’ Theorem as

$P\left( H | D \right)=\frac{P\left( D|H \right)P\left( H \right)}{P(D)}= \frac{P\left( D|H \right)P\left( H \right)}{\int P(D|H)P\left( H \right)dH}$ (5)

where $P\left( H | D \right)$ denotes a conditional posterior probability of the hypothesis (*H*) given the data $D$, $P\left( D | H \right)$ denotes the probability of observing the data *D* if the hypothesis *H* were true (also called the likelihood), and $P\left( H \right)$ indicates prior probability (degree of belief) of the hypothesis before being presented with more data. Calculation of the posterior probability of the hypothesis $P\left( H | D \right)$ requires dividing the likelihood of one hypothesis times its prior, $P\left( D | H \right)P\left( H \right)$, by $P(D)$ (Eq. 5, middle). When comparing many models, $P(D)$ becomes the integral over the likelihood times the prior probability for each hypothesis *H* within the set of all hypotheses $\mathcal{H}$ (Eq. 5, right).

When the hypothesis *H* in (Eq. 5) represents a candidate model *M* with *n* parameters, we can think of model *M* as the set of parameters, ***θ_M_*** = {*θ_1_, θ_2_, …, θ_n_*}. Each parameter *θ_i_* itself represents one dimension of the model’s probability space. Model optimization to data D assigns a likelihood to each ***θ_M_*** in probability space, and these values over the entire parameter space represent the numerator in (Eq. 5) with ${H=\boldsymbol{\theta}}_{\boldsymbol{M}}$, $P\left( D | \boldsymbol{\theta}_{M} \right)P(\boldsymbol{\theta}_{M})$, a distribution proportional to the probability distribution of model *M*. To calculate the probability distribution of *M*, we must also calculate the denominator of (Eq. 5), $\int P\left( D | \boldsymbol{\theta}_{M} \right)P\left( \boldsymbol{\theta}_{M} \right)d\boldsymbol{\theta}$, the so-called marginal likelihood or Bayesian evidence that normalizes the distribution of model *M* over all *n* parameters.

The marginal likelihood is often considered the average of the likelihood over the prior space (4–6). Consider a multidimensional parameter space for a model *M* where each point in space has an assigned likelihood. Then, if we add another parameter – another dimension – that is only mildly informative, the likelihood values barely change, and they are now spread across even more space. Thus, less evidence is present in the overall space, and therefore adding a parameter to a model will result in a lower marginal likelihood, unless that parameter allows the model *M* to fit the data *D* significantly better. In this way, the marginal likelihood intrinsically penalizes more complex models, unless a more complex model better matches the data (4–6).

**A.3** **AIC is calculated as an estimate of the Kullback-Liebler divergence**

The calculation of an information criterion is based on estimating the difference between candidate models and the “true” model (reality). The difference between statistical models or probabilities is measured by Kullback-Leibler (KL) divergence, represented by

$I\left( f,g \right)= \int f\left( x \right)ln(\frac{f(x)}{g(x|\boldsymbol{\theta})})dx$ (6)

where *f* and *g* represent probability distributions. In a typical KL divergence calculation, *f(x)* is the reference distribution, while *g(x)* is the distribution being compared. When using KL divergence in information theoretic model selection, as in (Eq. 6), we consider *f(x)* to be “reality,” while $g(x|\boldsymbol{\theta})$ is a candidate model parameterized by ***θ***, which are estimated from data *D* (as above). Here, $g(x|\boldsymbol{\theta})$ is a probability distribution of a model akin to the probability distribution of model *M*, $P(\boldsymbol{\theta}_{\boldsymbol{M}}\boldsymbol{|}D\boldsymbol{)}$ noted above. *I* represents information, and because *g* cannot exactly match reality, *I(f,g)* represents the information lost when approximating reality *f* by the model *g* (3).

The Akaike Information Criterion, or AIC, is an estimate of this KL divergence based on the likelihood function at its maximum point (1). It also considers the number of parameters in the model $g(x|\boldsymbol{\theta})$, which reduces bias in the estimate of the KL divergence for that model. Therefore, the AIC is denoted by

$\mathrm{AIC}=-2\ln\left( L\left( \boldsymbol{\theta}_{best} | D \right) \right)+2K= -2\ln\left( P\left( D | \boldsymbol{\theta}_{best} \right) \right)+2n$ (7)

where $L\left( \boldsymbol{\theta}_{best} | D \right)$ is the likelihood of the best-fitting parameter set and *K* is the number of parameters in the model (noted as $P\left( D | \boldsymbol{\theta}_{best} \right)$ and *n*, respectively, to correspond with the Bayesian definitions in **A.2**).

**A.4** **Notable differences between AIC and Bayesian evidence / posterior probability**

While they both aim to rank models from a set of candidates so that one or few can be chosen as the best models, there are several key differences between Bayesian evidence/posterior probability and AIC.

As noted in **A.2**, the Bayesian evidence or marginal likelihood is represented by $\int P\left( D | \boldsymbol{\theta}_{M} \right)P\left( \boldsymbol{\theta}_{M} \right)d\boldsymbol{\theta}$. Since model optimization to data *D* assigns a likelihood to each value over the entire parameter space, and is represented by $P\left( D | \boldsymbol{\theta}_{M} \right)P(\boldsymbol{\theta}_{M})$, integrating over this thereby integrates over every likelihood value across all evaluated parameter values in each dimension. In this way, the marginal likelihood takes into account every parameter set evaluated during optimization. This represents a more comprehensive score for a model compared to AIC, which as in (Eq. 7) only considers the best-fitting parameter set ***θ_best_***. Additionally, as noted with an example in **A.2**, the marginal likelihood penalizes for model complexity (number of parameters). AIC also includes the number of parameters *K* in its calculation as seen in (Eq. 7); however, this is again a less comprehensive means of penalizing for number of parameters compared to marginal likelihood.

We then consider the inclusion of prior expectations. At the level of model optimization, both AIC and marginal likelihood can be said to include prior expectations (depending on the algorithm used for optimization). Marginal likelihood includes prior expectations by default, represented by the $P\left( \boldsymbol{\theta}_{M} \right)$ term in the integral above. AIC requires only the best-fitting parameter set, so if optimization was performed via Bayesian principles where prior expectations for parameters were assigned, then these priors could well be said to affect ***θ_best_*** in the AIC calculation. However, Bayesian principles enable inclusion of prior expectations at the level of model ranking. Often, candidate models are considered equally likely a priori, (4–6) and will thus have a prior probability of $\frac{1}{n}$. When considering H in (Eq. 5) equal to a particular model *M_i_*, the posterior probability $P\left( M_{i} | D \right)$ firstly *requires* a prior probability $P(M_{i})$ for its calculation, and once calculated can be compared to that prior probability $\frac{1}{n}$. In this way, Bayesian principles provide a numerical metric to assess how data informed our knowledge about *M_i_*. This plays a role in model averaging as well (see **A.6**). AIC does not have a formal means for including prior probability in its calculation nor in the generation of a posterior probability. Probability in the use of AIC is an interpretation of relative likelihoods across candidate models and is not a true probability (see Eqs. 4 and 5 in the next section).

Given the incorporation of the full parameter space and prior expectations, as well as the ability to calculate a Bayesian probability that provides insight into knowledge gained from the data, we consider marginal likelihood the optimal means for model ranking and interpretation therein. A specific model selection example where AIC and marginal likelihood/posterior probabilities are compared is detailed in section **A.7**.

**A.5** **Model selection allows us to evaluate which variables or terms have the largest effect on observed data**

Model selection is the process of investigating candidate models, each of which may fit the data to a different extent, while prioritizing model simplicity (aka *lex parsimoniae* (7)). That is, the best model must comprise a balance between matching the data exactly and having the fewest variables and parameters required to do so. In linear regression, such as in the following example, this involves investigating which predictor variables with their fitted coefficients, best match the response variable (**Fig 1A**). In kinetic models, such as those with which we aim to capture the behavior of small cell lung cancer (SCLC), this involves investigating which variables and which model terms, with their fitted kinetic parameters, best match the data (**Fig 4B,C**).

For Bayesian model selection, the hypotheses $H$ in (Eq. 5) represent candidate models $M$ within the set of all models ℳ, and data *D* represents the dataset to which each model is fitted. Then, the likelihood of that model *M*, $P\left( D | M \right)$, is the marginal likelihood. Calculating the marginal likelihood for all candidate models and normalizing by the sum of this value for all models results in the probability of the model *M* given the data, $P\left( M | D \right)$. The higher the probability, the more likely the model best represents the process that generated the data (**Fig 1C**, black points).

Models can be ranked by AIC value alone, and in this case a lower AIC value indicates that a model is a better representation of the data. However, those using AIC can scale the models with respect to the minimum AIC value (1,3). This results in AIC differences,

$\Delta_{i}=AIC_{i}-min(AIC)$ (8)

where *min(AIC)* is the AIC value of the lowest-scoring (best) model candidate. Using AIC differences, Akaike weights are calculated,

$w_{i}=\frac{e^{-\frac{\Delta_{i}}{2}}}{\sum_{r=1}^{R} e^{-\frac{\Delta_{r}}{2}}}$ (9)

where *R* is the number of models in the candidate set. Akaike weights represent relative likelihoods of the models given the data, and are interpreted as probabilities (1). From this perspective, the higher the Akaike weight, the more likely that model best represents the process that generated the data (**Fig 1C**, red points).

**A.6** **Model averaging uses model selection outcomes from all models to demonstrate how the observed data informed the model variables or terms that represent our hypotheses**

Probabilities or weights are then used for model averaging. Model averaging is a process used when no clear single best model can be identified after model selection. Model averaging can reveal a model variable or term common to better-performing models, indicating that that the process represented by this variable or term is likely to play a role in the process that generated the data, and allowing the user to identify the parts of a model that best capture the data. In our application, this approach implies a departure from a deterministic single model to a probabilistic understanding of mechanisms, with the probabilities determined from the available data.

In Bayesian model averaging, (BMA) each model is weighted by its marginal likelihood or posterior probability (8) and the likelihood of model with particular term or variable can be used to generate an overall likelihood for that term or variable. Here, the prior probability of a term, which we typically consider to be equally likely vs. unlikely before model optimization, is important. Now, the *H* of (Eq. 5) indicates the hypothesis that a particular model term (individual process within the system of interest) is part of the “true” model, and the prior $P\left( H \right)$ and likelihood of that term $P\left( D|H \right)$ can be used to calculate $P\left( H | D \right)$. Thus we can investigate how the data informed our knowledge about *H*, from its prior to posterior probability.

To determine whether to predict that a model variable or term is likely to be involved in the system of interest, the Bayes Factor can be used. The Bayes Factor is the ratio of the likelihood of two hypotheses, and when prior probabilities are equal is equivalent to the ratio of the two posterior probabilities:

$BF= \frac{P\left( D | H_{1} \right)}{P\left( D | H_{2} \right)}= \frac{\frac{P\left( H_{1} | D \right)}{P\left( H_{1} \right)}}{\frac{P\left( H_{2} | D \right)}{P\left( H_{2} \right)}};BF= \frac{P\left( H_{1} | D \right)}{P\left( H_{2} | D \right)} when P\left( H_{1} \right)=P\left( H_{2} \right)$ (10)

Here, *H_1_* may represent the hypothesis that the model variable does play an important role, while *H_2_* may represent the hypothesis that it does not. The Bayes Factor (BF) between one hypothesis and another can be considered a true difference between the probability of each hypothesis when 10^-1/2^ > BF > 10^1/2^. The value of 10^1/2^ (~=3) is the lowest at which a difference may be determined; or 10^-1/2^ (~= ⅓) when the higher-probability hypothesis is in the denominator of the BF (9). In this way, comparing hypotheses via BF can result in one of three outcomes: $P(H_{1}|D)$ is > 3 times more likely than ${P(H}_{2}\left| D \right),$ meaning hypothesis *H_1_* is informed by the data to be significantly more likely than *H_2_*; $P(H_{1}|D)$ is < ⅓ times less likely than ${P(H}_{2}\left| D \right),$ meaning *H_1_* is informed by the data to be significantly less likely than *H_2_*; or, $P(H_{1}|D)$ is between ⅓ less likely and 3 times more likely than ${P(H}_{2}\left| D \right),$ meaning *H_1_* is not informed by the data to be significantly different than *H_2_*. We simply denote this last outcome as “*H_1_* is not informed by the data.”

When using information criteria such as AIC, parameter importance analysis is typically performed to investigate how well the data supports a model variable. For each variable, the Akaike weights of all models containing that variable are summed (1). The sum of weights (SW) is treated as the probability that a variable belongs in the “true” model, thus the biological feature represented by this variable plays an important role in the biological system. There does not appear to be an accepted threshold over which the SW for a variable indicates it should be included in a model, thus it seems practitioners choose a threshold on an ad hoc basis (10).

**A.7** **Advantages of Bayes-MMI over AIC for model selection and model averaging: continuing example**

Despite equivalent goals between BMA and SW, problems have been noted with the latter. To illustrate this point, we consider the example of (10), and compare AIC-based SW to our Bayesian method combining marginal likelihood results of model selection and BMA of terms across candidate models, named Bayes-MMI. In (10), investigators use data with four variables *x_1-4_* and one response variable *y*, similar to the example in Section **A.1,** where the farmer wishes to determine which animals (*x_1-4_*) most directly affect his income (*y*).

The investigators in (10) generated a “simulated ground truth” dataset where some predictor variables in *x_1-4_* would be clearly necessary to include in a representative model for the data, while others would be unnecessary:

“We simulated a data set (sample size *n=100*) including one response variable *y* and four predictor variables, *x_1_, x_2_, x_3_,* and *x_4_*. We controlled for the correlation structure both between the response variables and predictor variables and among predictor variables using a Cholesky decomposition (Genz & Bretz 2009). This method allows one predictor variable with a strong effect to be generated together with other variables with smaller tapering effects, as recommended by Burnham & Anderson (2002 p. 89, 2004).” (10)

Galipaud and colleagues provide R code in the Supporting Information of their article for their simulations, and we used their data.simulation method to generate a dataset with sample.size=100, and using the same tapering predictor variable effects, *r_y,x1_* = 0.70, *r_y,x1_* = 0.50, *r_y,x1_* = 0.05, *r_y,x1_* = 0.0. This generates a dataset of which a snippet is below:

|  | y | x1 | x2 | x3 | x4 |
| --- | --- | --- | --- | --- | --- |
| 1 | 5.927508 | 10.650031 | 9.644639 | 12.644369 | 9.585406 |
| 2 | 5.001812 | 10.255895 | 9.817701 | 9.236827 | 10.147333 |
| 3 | 5.540665 | 10.368334 | 11.221946 | 10.384068 | 10.537025 |
| … | … | … | … | … | … |
| 100 | 6.832196 | 11.740345 | 10.088608 | 11.213238 | 10.747818 |

In (10), Galipaud and colleagues performed model selection on simulated data using variable combinations including intercept-only, (16 models) calculating AICc (AIC corrected for small sample sizes) and assessed parameter importance using SW (10). Galipaud et al. used least squares regression via the R lm() function, while for our nested sampling parameter search we used a least squares likelihood function with PyMultinest searching the 5-dimensional parameter space (*β_1_*, *β_2_*, *β_3_*, *β_4_*, and *β_0_*) using uniform prior distributions of 0 to 10 for each response variable’s coefficient *β_1-4_* and -10 to 10 for the intercept *β_0_*. Our nested sampling results are similar to those in (10), as seen in **Table A**.

| **Table A. Summary of nested sampling model selection results on the simulated dataset and model selection problem in Galipaud et al., 2014, ranked by AICc.** | | | | | | | | | | | |
| --- | --- | --- | --- | --- | --- | --- | --- | --- | --- | --- | --- |
| ***β_0_*** | ***x_1_*** | ***x_2_*** | ***x_3_*** | ***x_4_*** | ***k*** | **rank, AICc** | **rank (AICc) in Galipaud et al. 2014** | ***log(L)*** | **AICc** | ***Δ_i_*** | ***w_i_*** |
| -2.502 | 0.608 | 0.139 |  |  | 4 | 0 | 0 | -48.764 | 103.778 | 0 | 0.313 |
| -3.398 | 0.618 | 0.129 | 0.089 |  | 5 | 1 | 1 | -47.728 | 103.877 | 0.099 | 0.298 |
| -2.729 | 0.620 | 0.141 |  | 0.007 | 5 | 2 | 2 | -48.776 | 105.973 | 2.195 | 0.105 |
| -3.329 | 0.619 | 0.131 | 0.077 | 0.002 | 6 | 3 | 3 | -47.739 | 106.115 | 2.338 | 0.097 |
| -2.169 | 0.623 |  | 0.090 |  | 4 | 4 | 5 | -50.264 | 106.778 | 3.001 | 0.070 |
| -1.219 | 0.617 |  |  |  | 3 | 5 | 4 | -51.362 | 106.848 | 3.070 | 0.068 |
| -1.462 | 0.615 |  |  | 0.025 | 4 | 6 | 6 | -51.299 | 108.847 | 5.069 | 0.025 |
| -2.300 | 0.614 |  | 0.086 | 0.026 | 5 | 7 | 7 | -50.241 | 108.903 | 5.125 | 0.024 |
| 3.357 |  | 0.164 |  |  | 3 | 8 | 8 | -96.251 | 196.625 | 92.847 | 10^-21^ |
| 2.990 |  | 0.164 | 0.038 |  | 4 | 9 | 9 | -96.061 | 198.372 | 94.594 | 10^-22^ |
| 3.355 |  | 0.164 |  | 0.001 | 4 | 10 | 11 | -96.255 | 198.761 | 94.983 | 10^-22^ |
| 3.043 |  | 0.163 | 0.029 | 0.004 | 5 | 11 | 13 | -96.088 | 200.597 | 96.819 | 10^-22^ |
| 4.990 |  |  |  |  | 2 | 12 | 10 | -100.00 | 202.041 | 98.263 | 10^-22^ |
| 4.602 |  |  | 0.039 |  | 3 | 13 | 12 | -99.787 | 203.698 | 99.920 | 10^-23^ |
| 4.901 |  |  |  | 0.009 | 3 | 14 | 14 | -99.991 | 204.106 | 100.328 | 10^-23^ |
| 4.510 |  |  | 0.041 | 0.007 | 4 | 15 | 15 | -99.784 | 205.818 | 102.041 | 10^-23^ |
| Candidate models are ranked by AICc as in Galipaud et al., 2014; rank in our analysis can be compared to the ranking in Galipaud et al., 2014 (“rank” *vs.* “rank in Galipaud et al., 2014” columns). Maximum log-likelihood parameter estimates and AICc are calculated from PyMultinest output. Parameter estimates are reported if present for each of the 16 candidate models. *k*, total number of estimable parameters; *log(L)*, maximum log-likelihood returned by our least-squares likelihood function; AICc, AIC “corrected” for small sample size; *Δ_i_*, *AICc – min(AICc)* per model; *w_i_*, Akaike weight. | | | | | | | | | | | |

As noted in the main text, AICc-based methods overestimate *x_4_*. According to AICc, $x_{4}$ can be found in the best-ranked models, while when ranking according to posterior probability, $x_{4}$ does not appear until the fifth-highest ranked model (**Figure 1C; Tables A and B**). In fact, $x_{4}$ appears only in candidate models whose posterior probability has *decreased* compared to the prior probability (**Figure 1C,** dotted lines above vs below posterior probability points; **Table B**). The four highest-ranked models have a cumulative probability of 0.916 (**Figure 1C;** **Table B**). Thus, most of the probability that one candidate model is the best model is contained in those models, which more accurately do not include $x_{4}$. As in the main text, we also compare prior probability of variable inclusion in the “true” model (0.5, or equivalent between presence and absence) to posterior probabilities based on marginal likelihood (**Fig 1D, Table C**).

| **Table B. Summary of nested sampling model selection results on the simulated dataset and model selection problem in Galipaud et al., 2014, ranked by posterior probability.** | | | | | | | | | | | | |
| --- | --- | --- | --- | --- | --- | --- | --- | --- | --- | --- | --- | --- |
| ***β_0_*** | ***x_1_*** | ***x_2_*** | ***x_3_*** | ***x_4_*** | ***k*** | **rank, post. prob.** | **rank, AICc** | **rank (AICc) in Galipaud et al. 2014** | ***log(Z)*** | ***log(Z)* error (+/-)** | **prior prob.** | **post. prob.** |
| -2.502 | 0.608 | 0.139 |  |  | 4 | 0 | 0 | 0 | -57.28 | 0.049 | 0.062 | 0.448 |
| -1.219 | 0.617 |  |  |  | 3 | 1 | 5 | 4 | -58.03 | 0.043 | 0.062 | 0.212 |
| -3.398 | 0.618 | 0.129 | 0.089 |  | 5 | 2 | 1 | 1 | -58.25 | 0.054 | 0.062 | 0.170 |
| -2.169 | 0.623 |  | 0.090 |  | 4 | 3 | 4 | 5 | -58.94 | 0.049 | 0.062 | 0.086 |
| -2.729 | 0.620 | 0.141 |  | 0.007 | 5 | 4 | 2 | 2 | -59.75 | 0.055 | 0.062 | 0.038 |
| -1.462 | 0.615 |  |  | 0.025 | 4 | 5 | 6 | 6 | -60.24 | 0.050 | 0.062 | 0.023 |
| -3.329 | 0.619 | 0.131 | 0.077 | 0.002 | 6 | 6 | 3 | 3 | -60.72 | 0.060 | 0.062 | 0.014 |
| -2.300 | 0.614 |  | 0.086 | 0.026 | 5 | 7 | 7 | 7 | -61.06 | 0.055 | 0.062 | 0.010 |
| 3.357 |  | 0.164 |  |  | 3 | 8 | 8 | 8 | -102.99 | 0.044 | 0.062 | 10^-21^ |
| 4.990 |  |  |  |  | 2 | 9 | 12 | 10 | -104.79 | 0.039 | 0.062 | 10^-21^ |
| 2.990 |  | 0.164 | 0.038 |  | 4 | 10 | 9 | 9 | -104.86 | 0.050 | 0.062 | 10^-22^ |
| 3.355 |  | 0.164 |  | 0.001 | 4 | 11 | 10 | 11 | -105.64 | 0.051 | 0.062 | 10^-22^ |
| 4.602 |  |  | 0.039 |  | 3 | 12 | 13 | 12 | -106.78 | 0.045 | 0.062 | 10^-22^ |
| 4.901 |  |  |  | 0.009 | 3 | 13 | 14 | 14 | -107.27 | 0.046 | 0.062 | 10^-23^ |
| 3.043 |  | 0.163 | 0.029 | 0.004 | 5 | 14 | 11 | 13 | -107.64 | 0.057 | 0.062 | 10^-23^ |
| 4.510 |  |  | 0.041 | 0.007 | 4 | 15 | 15 | 15 | -109.26 | 0.052 | 0.062 | 10^-23^ |
| Candidate models are ranked by posterior probability (“post. prob.”). Ranking in this Bayesian analysis can be compared to ranking via AICc in this analysis and to the ranking in Galipaud et al., 2014 (“rank, post. prob.”, *vs.* “rank, AICc” *vs.* “rank (AICc) in Galipaud et al., 2014” columns). First five columns of maximum log-likelihood parameter estimates are part of PyMultinest output. Parameter estimates are reported if present for each of the 16 candidate models. *k*, total number of estimable parameters; *log(Z)*, the natural log of the Bayesian evidence/marginal likelihood (*Z*), calculated within the prior-bounded parameter space using our least-squares likelihood function; *log(Z) error*, the error returned by PyMultinest; prior prob., the prior probability that a model is the “correct” model; post. prob., posterior probability that the model is “correct”, calculated as $\frac{Z_{i}*P\left( i \right)}{\sum_{j} Z_{j}*P\left( j \right)}$. | | | | | | | | | | | | |

If *H_1_* represents “*x_4_* should be included in the model”, and *H_2_* the opposite, the BF for *H_1_* vs *H_2_* is 0.07/(1-0.07) = .075 < ⅓, and we determine that *x_4_* should not be included in the model. The case of *x_3_* is also instructive, where the prior probability of inclusion at 0.5 has changed to a posterior probability of 0.28 after incorporation of data. With the very weak correlation of *x_3_* to *y* at 0.05, Bayesian inference has indicated that, at least with the data used, a definitive choice to include or exclude *x_3_* can almost, though not quite, be made (0.28/(1-0.28) = 0.389 > ⅓). The SW for *x_3_* is 0.49, and as such a practitioner certainly might include it in a chosen model, possibly without considering that such a weakly correlated variable could achieve this SW.

| **Table C. SW and posterior probability calculations for each model variable in both full candidate set and partial candidate set examples.** | | | | | | |
| --- | --- | --- | --- | --- | --- | --- |
| **Variable** | **SW: Galipaud et al. 2014** | **SW: this manuscript** | **SW: candidate subset** | **Prior probability** | **Posterior probability** | **Post prob: candidate subset** |
| ***x_1_*** | 1 | 1 | 1 | 0.5 | 1 | 1 |
| ***x_2_*** | 0.94 | 0.81 | 0.82 | 0.5 | 0.67 | 0.67 |
| ***x_3_*** | 0.37 | 0.49 | 0.42 | 0.5 | 0.28 | 0.42 |
| ***x_4_*** | 0.37 | 0.25 | 0.15 | 0.5 | 0.09 | 0.12 |
| Prior probability for a variable is set at 0.5, meaning a variable’s prior probability for can be calculated per candidate model by dividing 0.5 by the number of models in which the variable appears. Prior probability values only impact posterior probability scores and not SW calculations. | | | | | | |

**Note B.** **Sums of AIC weights and posterior probability on a subset of the candidate models.**

An important feature of SW is that it is most accurately calculated when the full candidate model set contains equal representation of every variable (1). That is, *x_1_* if appears in 8 models, *x_2_*, *x_3_*, and *x_4_* must appear in 8 models to calculate an SW for each of them. That is the case in this example from (10). However, we were interested in the case where not every variable may be represented equally in the candidate model set. Once 10 variables are present, a candidate set including equal representation of every variable would include more than 1000 models; more than 13 variables means the full candidate set includes more than 10,000 models. When many variables are present, it is possible to eliminate models from a candidate set, achieving a more tractable number of candidate models, using prior knowledge. We chose to impose synthetic prior knowledge on this 16-model candidate set, as if it is “known” that *x_3_* never appears along with *x_4_*. Removing every model that includes both *x_3_* and *x_4_* left us with 12 models rather than 16, where *x_1_* and *x_2_* appeared in 6 models each, while *x_3_* and *x_4_* appeared in 4 models each.

We repeated our analysis on this subset of candidate models (**Table D**). Similar to our Bayesian analysis of the entire set of candidate models, *x_4_* does not appear until the fifth-highest ranked model; the four highest-ranked models having a cumulative probability of 0.938 (the majority of probability that the “true” model is present); *x_4_* still appears only in candidate models whose posterior probability has decreased compared to the prior (**Table D**). The SW for *x_4_* in this analysis is 0.14, having changed from its SW of 0.25 using the whole candidate set (**Table C**). There does not appear to be an accepted threshold over which the SW for a variable indicates it should be included in a model, thus it seems practitioners choose a threshold on a per-case basis (10). As such, it is difficult to assess what a change in SW from 0.25 to 0.14 means when using only a subset of the data – as noted, however, this is not an appropriate use for SWs

| **Table D. Summary of AICc and nested sampling model selection results using a partial candidate set.** | | | | | | | | | | | |
| --- | --- | --- | --- | --- | --- | --- | --- | --- | --- | --- | --- |
| **Model** | ***log(L)*** | **AICc** | ***Δ_i_*** | ***w_i_*** | **Rank, post. prob.** | **Rank, AICc** | ***log(Z)*** | ***log(Z)* error** | | **Prior prob** | **Post prob** |
| $y=x_{1}+x_{2}+\beta$ | -48.764 | 103.78 | 0 | 0.357 | 0 | 0 | -57.280 | 0.048 | 0.083 | | 0.459 |
| $y=x_{1}+\beta$ | -51.362 | 106.85 | 3.070 | 0.077 | 1 | 4 | -58.028 | 0.043 | 0.083 | | 0.217 |
| $y=x_{1}+x_{2}+x_{3}+\beta$ | -47.728 | 103.88 | 0.099 | 0.340 | 2 | 1 | -58.250 | 0.054 | 0.083 | | 0.174 |
| $y=x_{1}+x_{3}+\beta$ | -50.264 | 106.78 | 3.001 | 0.080 | 3 | 3 | -58.936 | 0.049 | 0.083 | | 0.088 |
| $y=x_{1}+x_{2}+x_{4}+\beta$ | -48.776 | 105.97 | 2.195 | 0.119 | 4 | 2 | -59.751 | 0.055 | 0.083 | | 0.039 |
| $y=x_{1}+x_{4}+\beta$ | -51.299 | 108.85 | 5.069 | 0.028 | 5 | 5 | -60.243 | 0.050 | 0.083 | | 0.024 |
| $y=x_{2}+\beta$ | -96.251 | 196.63 | 92.85 | 10^-21^ | 6 | 6 | -102.989 | 0.044 | 0.083 | | 10^-21^ |
| $y=\beta$ | -100.00 | 202.04 | 98.26 | 10^-22^ | 7 | 9 | -104.787 | 0.038 | 0.083 | | 10^-21^ |
| $y=x_{2}+x_{3}+\beta$ | -96.061 | 198.37 | 94.59 | 10^-21^ | 8 | 7 | -104.864 | 0.050 | 0.083 | | 10^-21^ |
| $y=x_{2}+x_{4}+\beta$ | -96.255 | 198.76 | 94.98 | 10^-22^ | 9 | 8 | -105.643 | 0.051 | 0.083 | | 10^-21^ |
| $y=x_{3}+\beta$ | -99.787 | 203.70 | 99.92 | 10^-23^ | 10 | 10 | -106.782 | 0.045 | 0.083 | | 10^-21^ |
| $y=x_{4}+\beta$ | -99.991 | 204.11 | 100.3 | 10^-23^ | 11 | 11 | -107.266 | 0.046 | 0.083 | | 10^-21^ |
| Candidate models are ranked by posterior probability (“post. prob.”). Ranking in this Bayesian analysis can be compared to ranking via AICc in this analysis (“rank, post. prob.”, *vs.* “rank, AICc”). Both AICc-related calculations (second through fifth column) and Bayesian calculations (eight through final column) are shown for the partial candidate set results. *log(L)*, maximum log-likelihood; AICc, AIC “corrected” for small sample size; *Δ_i_*, *AICc – min(AICc)* per model; *w_i_*, Akaike weight; *log(Z)*, the natural log of the Bayesian evidence/marginal likelihood (*Z*); *log(Z) error*, the error returned by PyMultinest; prior prob., the prior probability that a model is the “correct” model; post. prob., posterior probability that the model is “correct”, calculated as $\frac{Z_{i}*P\left( i \right)}{\sum_{j} Z_{j}*P\left( j \right)}$. | | | | | | | | | | | |

anyway (1).

For our Bayesian analysis, the posterior probability that the inclusion of *x_4_* is supported by the data is 0.09, (**Table C**) and is similar for this full candidate set and for the “prior knowledge excluded” candidate set (0.09 vs 0.12). Here, *x_4_* is just as unlikely to be included in the “true” model whether assessing a subset or the full set of candidate models. Interestingly, the posterior probability of *x_3_* changes its numerical value when analyzing a subset of the data: from 0.28 in the full candidate set to 0.42 in the “prior knowledge excluded” partial candidate set. However, Bayesian principles dictate that we *can* assess if this is a significant change. With equal prior probability per model variable (**Table C**), a posterior probability of 0.75 or more, or probability of 0.25 or less, would be considered substantial evidence for inclusion or exclusion of that variable, respectively ((9); see **A.6**). Thus, the change from 0.28 to 0.64 remains in the region between 0.25 and 0.75 where we would consider the data not to have informed whether to include *x_3_* in the model. All other variables (*x_1_*, *x_2_*, *x_4_*) remained within 0.03 of their full-set values and thus remained either in the “informed and should be included” set (*x_1_*, *x_2_*) or the “informed and should be excluded” set (*x_4_*). It is likely that the weak correlation of *x_3_* with the response variable resulted in the Bayesian analysis result of “unsure” whether *x_3_* should be included.

**Note C. Bayesian Information Criterion**

The theory of Bayesian information criterion (BIC), or Schwarz Information Criterion, is grounded in a relationship to the Bayes Factor (BF) (Eq. 10); a BF comparing two candidate models’ marginal likelihoods has been shown to be equivalent to comparing those models’ BIC values (11). BIC is calculated as,

$\mathrm{BIC}=-2\ln\left( L\left( \boldsymbol{\theta}_{best} | D \right) \right)+k*ln(n)$, (11)

where *k* is the number of parameters in the candidate model, *n* is the number of data points in the observed data, and $L\left( \boldsymbol{\theta}_{best} | D \right)$ is the likelihood of the best-fitting parameter set (11,12).

BIC was later shown to be derivable from the posterior probability of a candidate model assuming equal candidate model priors, asymptotically approximating -2*ln(P(*M_k_|D*)) for the *k*^th^ candidate model (12). Thus, the marginal likelihood can be approximated by

$P\left( M_{k} | D \right)\propto P\left( D | M_{k} \right)P\left( M_{k} \right)\approx e^{-\frac{{BIC}_{k}}{2}}P(M_{k})$, (12)

or

$P\left( D | M_{k} \right)\approx e^{-\frac{{BIC}_{k}}{2}}$, (13)

given that candidate prior probabilities are equal across candidate models.

BIC is similar to AIC in that it requires the number of parameters, data points, and only one likelihood value from candidate model fitting. It is derivable from the marginal likelihood of a candidate model and in this way is perhaps closer to the marginal likelihood than AIC is. The general behavior of BIC is that it penalizes number of parameters in a candidate model more strongly than AIC (11).

Galipaud and colleagues evaluate BIC behavior in contrast with AIC behavior in their variable importance example, finding it a generally more accurate measure of variable importance, but only in very large sample sizes (10). We calculated BIC on the (10) simulated dataset, and used the marginal likelihood approximation to calculate posterior probability across candidate models via

$P(M_{k}|D)\approx\frac{e^{-\frac{{BIC}_{k}}{2}}}{\sum_{r=1}^{R} e^{-\frac{{BIC}_{r}}{2}}}$ , (14)

where *R* is the total number of candidate models.

| **Table E. Summary of nested sampling model selection results on the simulated dataset and model selection problem in Galipaud et al., 2014, ranked by BIC-estimated posterior probability.** | | | | | | | | | | | | | |
| --- | --- | --- | --- | --- | --- | --- | --- | --- | --- | --- | --- | --- | --- |
| ***β_0_*** | ***x_1_*** | ***x_2_*** | ***x_3_*** | ***x_4_*** | ***k*** | **rank, BIC post. prob.** | **Rank, post. prob.** | | ***Log(L)*** | **BIC** | $-\frac{{BIC}_{k}}{2}$ | **prior prob.** | **BIC post. prob.** |
| -2.502 | 0.608 | 0.139 |  |  | 4 | 0 | 0 | -48.76 | | 111.34 | -55.67 | 0.062 | 0.404 |
| -1.219 | 0.617 |  |  |  | 3 | 1 | 1 | -51.36 | | 111.94 | -55.97 | 0.062 | 0.300 |
| -3.398 | 0.618 | 0.129 | 0.089 |  | 5 | 2 | 2 | -47.73 | | 113.88 | -56.94 | 0.062 | 0.114 |
| -2.169 | 0.623 |  | 0.090 |  | 4 | 3 | 3 | -50.26 | | 114.34 | -57.17 | 0.062 | 0.090 |
| -2.729 | 0.620 | 0.141 |  | 0.007 | 5 | 4 | 4 | -48.78 | | 115.97 | -57.99 | 0.062 | 0.040 |
| -1.462 | 0.615 |  |  | 0.025 | 4 | 5 | 5 | -51.30 | | 116.41 | -58.21 | 0.062 | 0.032 |
| -3.329 | 0.619 | 0.131 | 0.077 | 0.002 | 6 | 6 | 6 | -47.74 | | 118.50 | -59.25 | 0.062 | 0.011 |
| -2.300 | 0.614 |  | 0.086 | 0.026 | 5 | 7 | 7 | -50.24 | | 118.90 | -59.45 | 0.062 | 0.009 |
| 3.357 |  | 0.164 |  |  | 3 | 8 | 8 | -96.25 | | 201.71 | -100.86 | 0.062 | 10^-21^ |
| 4.990 |  |  |  |  | 2 | 9 | 9 | -100.00 | | 204.61 | -102.30 | 0.062 | 10^-21^ |
| 2.990 |  | 0.164 | 0.038 |  | 4 | 10 | 10 | -96.06 | | 205.94 | -102.97 | 0.062 | 10^-21^ |
| 3.355 |  | 0.164 |  | 0.001 | 4 | 11 | 11 | -96.26 | | 206.33 | -103.16 | 0.062 | 10^-22^ |
| 4.602 |  |  | 0.039 |  | 3 | 12 | 12 | -99.79 | | 208.79 | -104.39 | 0.062 | 10^-22^ |
| 4.901 |  |  |  | 0.009 | 3 | 13 | 13 | -99.99 | | 209.19 | -104.60 | 0.062 | 10^-22^ |
| 3.043 |  | 0.163 | 0.029 | 0.004 | 5 | 14 | 14 | -96.09 | | 210.60 | -105.30 | 0.062 | 10^-22^ |
| 4.510 |  |  | 0.041 | 0.007 | 4 | 15 | 15 | -99.78 | | 213.39 | -106.69 | 0.062 | 10^-23^ |
| Candidate models are ranked by BIC-estimated posterior probability (“BIC post. prob.”). Ranking in this BIC analysis can be compared to ranking via nested-sampling derived posterior probability (“rank, BIC post. prob.”, *vs.* “rank, post. prob.” columns). First five columns of maximum log-likelihood parameter estimates are part of PyMultinest output. Parameter estimates are reported if present for each of the 16 candidate models. *k*, total number of estimable parameters; *log(L)*, maximum log-likelihood returned by our least-squares likelihood function;  $-\frac{{BIC}_{k}}{2}$, since $e^{-\frac{{BIC}_{k}}{2}}$ (see Eq. 13) is the estimated marginal likelihood (Z) from BIC, we use $log(e^{-\frac{{BIC}_{k}}{2}})$ here to correspond to *log(Z)* as used in **Table B**; prior prob., the prior probability that a model is the “correct” model; post. prob., posterior probability that the model is “correct”, calculated for BIC estimate as $\frac{e^{-\frac{{BIC}_{k}}{2}}}{\sum_{r=1}^{R} e^{-\frac{{BIC}_{r}}{2}}} .$ | | | | | | | | | | | | | |

We found that these posterior probability estimates were closer to marginal likelihood posterior probability calculations than to AICc weights (**Table E**), and that the correlation coefficient between marginal likelihood posterior probability and BIC-estimated posterior probability was higher than between marginal likelihood posterior probability and AICc weights (**Fig S1**).

As an approximation of the marginal likelihood, *e*^-^*^BIC^*^/2^ can be multiplied by prior probabilities to calculate posterior probability of variable inclusion in the model in both the full candidate model set (**Note A.6**) and the candidate subset (**Note B**). In these assessments, BIC performed similarly to marginal likelihood (**Table F**).

| **Table F. Comparing BIC-estimated probability and marginal likelihood-calculated probability for each model variable in full and partial candidate sets.** | | | | | |
| --- | --- | --- | --- | --- | --- |
| **Variable** | **Prior probability** | **BIC-estimated probability: full** | **BIC-estimated prob: candidate subset** | **Posterior probability: full** | **Post prob: candidate subset** |
| ***x_1_*** | 0.5 | 1 | 1 | 1 | 1 |
| ***x_2_*** | 0.5 | 0.57 | 0.57 | 0.67 | 0.67 |
| ***x_3_*** | 0.5 | 0.22 | 0.34 | 0.28 | 0.42 |
| ***x_4_*** | 0.5 | 0.09 | 0.14 | 0.09 | 0.12 |
| Prior probability for a variable is set at 0.5, meaning a variable’s prior probability for can be calculated per candidate model by dividing 0.5 by the number of models in which the variable appears. | | | | | |

Given the similarities between BIC and marginal likelihood in this example, we compared AIC, BIC, and marginal likelihood values on the SCLC candidate models compared to each experimental dataset (**Fig 4**). However, the correlation between marginal likelihood and BIC or AIC using the SCLC data and candidate models was very dissimilar compared to the example from (10) described in **Note A.7** (**Fig S1**).

The main difference between the marginal likelihood and BIC or AIC is that the marginal likelihood calculation (from nested sampling) takes into account all parameter likelihood values from the evaluated parameter space for fitting each candidate model, and incorporates these into the marginal likelihood calculation (4–6); BIC and AIC use one likelihood. With a complex parameter space, or even one with multimodal best-fitting-parameter maxima (4–6), marginal likelihood is likely to incorporate such information into its value while this would be lost in the BIC and AIC values.

We hypothesize that the parameter space inherent in the (10) example dataset and candidate models is much simpler than the SCLC problem addressed here, resulting in the close correlation between marginal likelihood, AIC, and BIC in this case; the added complexity in the SCLC problem results in very different outcomes. Therefore, in complex problems, we consider the marginal likelihood to provide more informative values for comparing models.

References

1. Burnham KP&, Anderson DR. Model Selection and Multimodel Inference: a Practical Information‐theoretic Approach. 2nd ed. Springer, New York. 2002.

2. Hinne M, Gronau QF, van den Bergh D, Wagenmakers EJ. A Conceptual Introduction to Bayesian Model Averaging. Adv Methods Pract Psychol Sci. 2020;3(2):200–15.

3. Portet S. A primer on model selection using the Akaike Information Criterion. Infect Dis Model [Internet]. 2020 Jan 1 [cited 2022 Dec 4];5:111. Available from: /pmc/articles/PMC6962709/

4. Feroz F, Hobson MP. Multimodal nested sampling: an efficient and robust alternative to Markov Chain Monte Carlo methods for astronomical data analyses. Mon Not R Astron Soc [Internet]. 2008 Feb 21 [cited 2021 Nov 3];384(2):449–63. Available from: https://academic.oup.com/mnras/article/384/2/449/1023701

5. Feroz F, Hobson MP, Bridges M. MultiNest: an efficient and robust Bayesian inference tool for cosmology and particle physics. Mon Not R Astron Soc [Internet]. 2009 Oct 1 [cited 2021 Nov 3];398(4):1601–14. Available from: https://academic.oup.com/mnras/article/398/4/1601/981502

6. Feroz F, Hobson MP, Cameron E, Pettitt AN. Importance Nested Sampling and the MultiNest Algorithm. The Open Journal of Astrophysics [Internet]. 2019 Nov 27 [cited 2021 Nov 3];2(1):11120. Available from: https://github.com/farhanferoz/MultiNest.

7. Wildner M. In memory of William of Occam. Lancet [Internet]. 1999 Dec 18 [cited 2022 Dec 13];354(9196):2172. Available from: http://www.thelancet.com/article/S0140673605770859/fulltext

8. Fragoso TM, Bertoli W, Louzada F. Bayesian Model Averaging: A Systematic Review and Conceptual Classification. International Statistical Review [Internet]. 2018 Apr 1 [cited 2023 Jan 30];86(1):1–28. Available from: https://onlinelibrary-wiley-com.proxy.library.vanderbilt.edu/doi/full/10.1111/insr.12243

9. Kass RE, Raftery AE. Bayes Factors. J Am Stat Assoc. 1995;90(430):773–95.

10. Galipaud M, Gillingham MAF, David M, Dechaume-Moncharmont FX. Ecologists overestimate the importance of predictor variables in model averaging: a plea for cautious interpretations. O’Hara RB, editor. Methods Ecol Evol [Internet]. 2014 Oct 1 [cited 2021 Apr 25];5(10):983–91. Available from: http://doi.wiley.com/10.1111/2041-210X.12251

11. Dziak, JJ, Coffman, DL, Lanza, ST, Li, R, Jermiin, LS. Sensitivity and specificity of information criteria. *Briefings in Bioinformatics*. 2020. *21*(2): 553–565. Available from: <https://doi.org/10.1093/BIB/BBZ016>

12. Neath, AA, Cavanaugh, JE. The Bayesian information criterion: background, derivation, and applications. *Wiley Interdisciplinary Reviews: Computational Statistics*. 2012. 4: 199–203. Available from: <https://doi.org/10.1002/WICS.199>
