# Supplementary material for: Unified tumor growth mechanisms from multimodel inference and dataset integration
Source: PLoS Comput Biol. 2023 Jul 5;19(7):e1011215. doi: 10.1371/journal.pcbi.1011215 (PMC10351715; doi:10.1371/journal.pcbi.1011215)
Supplement: S2 Text — Note A. Population dynamics modeling and inter-subtype effects. Note B. Ordinary differential equations representing each SCLC subtype in the population dynamics models. (DOCX) [file pcbi.1011215.s002.docx]

**S2 Text. Population dynamics modeling of small cell lung cancer**

**Note A. Population dynamics modeling and inter-subtype effects**


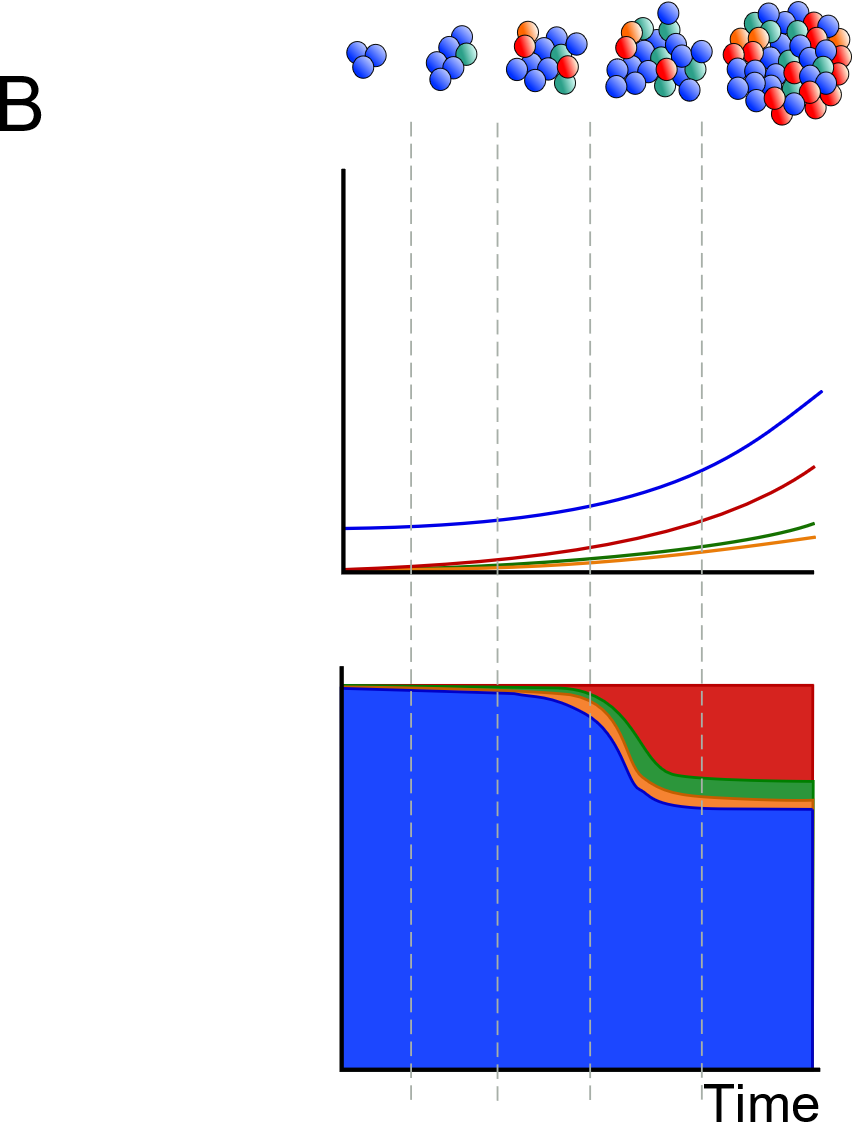

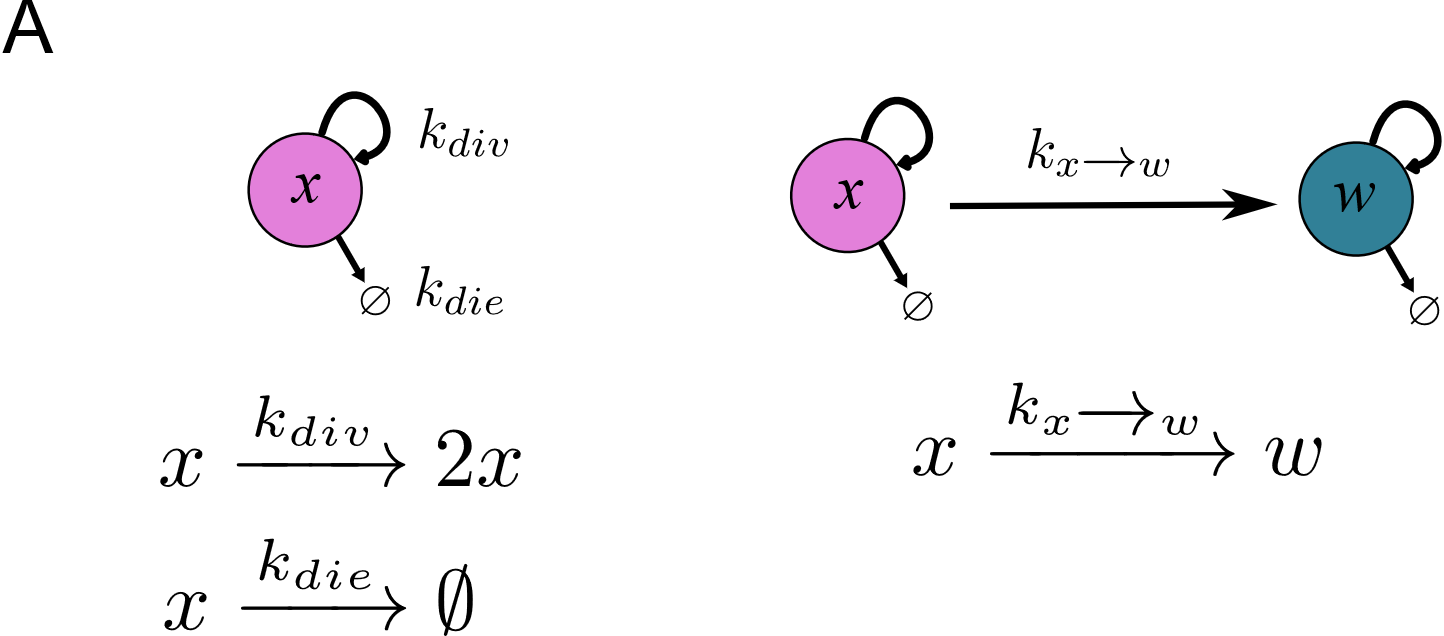
A population dynamics model represents behaviors of species over time, tracking the size of a cell population, rather than tracking individual cells. Such a model can also include signaling and dependence between species (1). Population changes are represented as reactions, where subpopulation abundances increase or decreases due to varying events (division, death, phenotypic transitions), and the rates of increase or decrease can be affected by the presence of other subpopulations. Here, cell types *x* and *w* undergo division, death and phenotypic transition at rates *k_div_*, *k_die_*, and *k_x-w_*, respectively (A).

In our case, where species are cells of an SCLC tumor population,

each is assigned a subtype identity (A, N, A2, or Y). The model

simulates tumor growth over time, (B, top) calculating increase in

subpopulation amount (B, middle), from which tumor subtype proportion is calculated (B, bottom) and compared at steady state to tumor proportion data. We define steady state as the tumor composition based on relative abundance of each cell subtype in the tumor, without external perturbations. Tumor growth may continue exponentially in steady-state, but subpopulation proportions within remain constant (1).

Non-spatial cell-cell interactions may also be modeled, by changing the rates of reactions for one subpopulation according to the amount of another subpopulation present. Here, we use the hypothesized biology where subtype *w* produces a secreted signal *f* that affects subtype *x*.

Our model is agnostic to the identity of *f*, but as a secreted signal we consider it an exosome or cytokine. We make the assumption that *f* may affect multiple cells *x*, as exosomes can directly bind to cells and also may undergo multiple cell uptake and release cycles (2). With these assumptions and chemical kinetic-style equations representing them, we calculate the change in reaction rates. In (C), *w* secretes the unknown signaling factor *f* at a rate of *k_f_*, while *f* is degraded at rate *k_-f_*. Factor *f* may affect subtype *x* at a rate of *k_e_*, where *x* becomes *x** (“*x* under effect of *f*”). Subtype *x* can then revert back to its unaffected form at a rate of *k_-e_*, indicating the rate at which the signaling is completed.


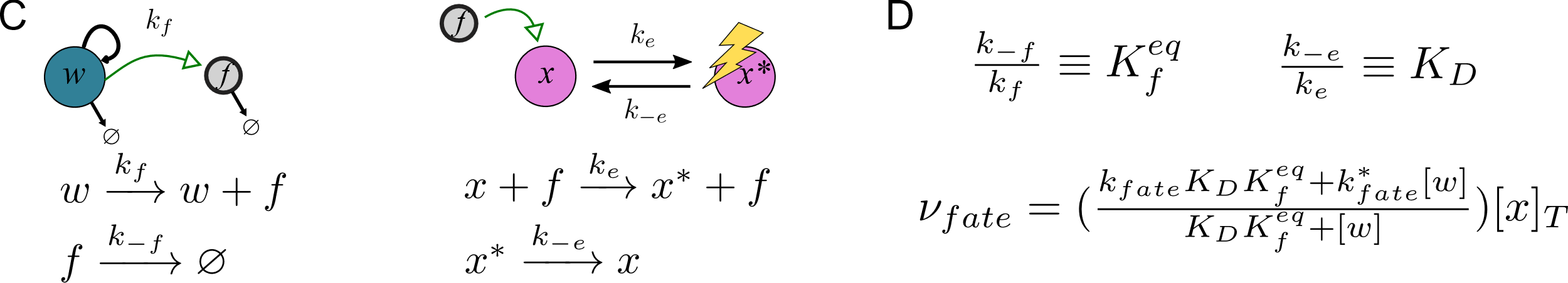


With *k_f_* the production rate constant and *k_-f_* the degradation rate constant, *K_f_^eq^* is the equilibrium constant for the amount of factor *f* in the system (D). Similarly, *K_D_* is the equilibrium constant related to the on-effect rate constant *k_e_* and the off-effect rate constant *k_-e_*. Utilizing the quasi-steady-state assumption to consider rates of production and degradation of *f* as approximately equal, and the partial equilibrium assumption to consider rates of conversion between affected cell *x** and unaffected cell *x* approximately equal, an analytical expression can be derived to calculate the rate of a cell fate (division, death, or phenotypic transition) for *x* (*ν_fate_*) as a function of the population size of the effector cell *w* (D) (1).

By assigning the value of *k^*^_fate_* as more or less than *k_fate_* for a particular cell fate, the presence of the effector cell subpopulation can increase or decrease, respectively, the rate of the cell fate for subpopulation *x*. In our population dynamics model, typically effector cells increase division and transition rates and decrease death rates, as shown in (E).


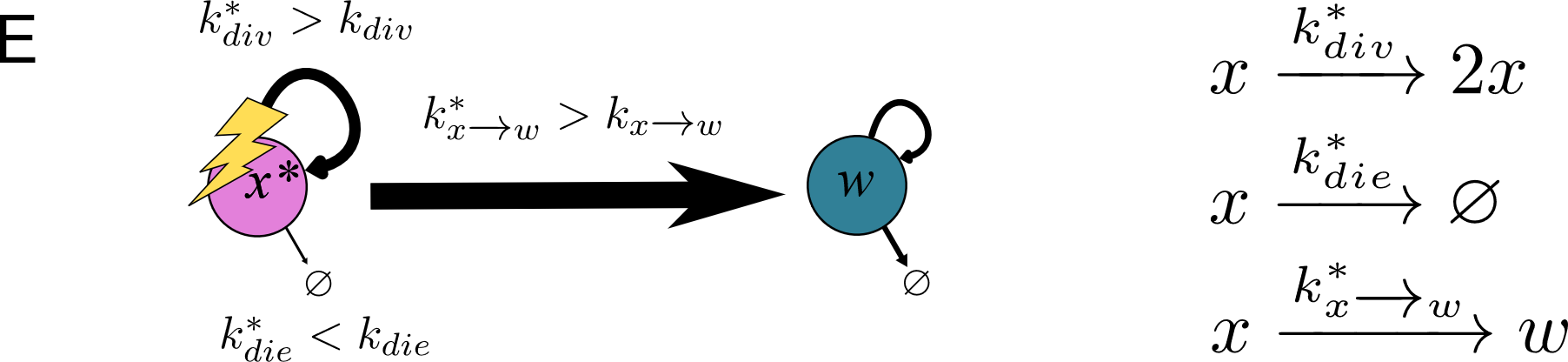


**Note B. Ordinary differential equations representing each SCLC subtype in the population dynamics models.**

Because we perform model selection, the ordinary differential equation (ODE) for each SCLC subtype may not be the same in each model. Here we provide an ODE per subtype that incorporates the most possible terms. These are color-coded to indicate when a term will be present in the ODE based on the features of the candidate model.

Subtype A:

$$\frac{dA}{dt}=\left( \frac{k_{{div}_{A}}K_{{div}_{A}}^{eq.assn}+{k_{{div}_{A}}^{*}[{effector}_{div}]}_{T}}{K_{{div}_{A}}^{eq.assn}+{[{effector}_{div}]}_{T}}-\frac{k_{{die}_{A}}K_{{die}_{A}}^{eq.assn}+{k_{{die}_{A}}^{*}\left[ {effector}_{div} \right]}_{T}}{K_{{die}_{A}}^{eq.assn}+\left[ {effector}_{div} \right]_{T}}-\frac{k_{A\to N}K_{A\to N}^{eq.assn}+{k_{A\to N}^{*}\left[ {effector}_{tsn} \right]}_{T}}{K_{A\to N}^{eq.assn}+\left[ {effector}_{tsn} \right]_{T}}-\frac{k_{A\to A2}K_{A\to A2}^{eq.assn}+{k_{A\to A2}^{*}\left[ {effector}_{tsn} \right]}_{T}}{K_{A\to A2}^{eq.assn}+\left[ {effector}_{tsn} \right]_{T}}-k_{A\to Y} \right){[A]}_{T}+k_{N\to A}{[N]}_{T}+k_{A2\to A}{[A2]}_{T}+k_{Y\to A}{[Y]}_{T}$$

[*effector_div_*], [*effector_tsn_*]: each can be either [Y] or [A2] + [Y]

: if effects on division and death are present

: if effects on transitions are present

: if the A→N and A→A2 transitions are present

: if the N→A and A2→A transitions are present

: if the A→Y transition is present

: if the Y→A transition is present

$K_{param}^{eq.assn}$represents *K_D_K_f_^eq^* for rate of subtype behavior represented by *param*. As stated in **Note A**, factor *f* and its interactions with each subtype are considered only within a simple motif in order to derive an analytical expression for how an action performed by a subtype is affected. Thus, *f* and its interactions are not explicitly modeled, but rather represented in each ODE via the combined expression in **Note A.D**. Because each action (division, death, transition toward a specific subtype) might be affected uniquely, *K_D_K_f_^eq^* for the rate of that action – for that parameter – is fit separately per parameter. See **Fig S3** indicating that per parameter, there is a baseline rate (e.g., division_A_baseline indicating *k_div_A_*), a rate if affected (e.g., division_A_altered indicating *k*_div_A_*) and the combined equilibrium constant for that parameter (e.g., division_A_equil_assn indicating *K_D_K_f_^eq^* for affected A division, or $K_{{div}_{A}}^{eq.assn}$).

Because analytically deriving the equation representing subtype fate enables the use of total subtype amount regardless of whether it is affected or unaffected (e.g., [A]_T_ rather than [A]+[A*]), all ODEs use only the total amount of subtype.

Subtype N:

$$\frac{dN}{dt}=\left( \frac{k_{{div}_{N}}K_{{div}_{N}}^{eq.assn}+{k_{{div}_{N}}^{*}[{effector}_{div}]}_{T}}{K_{{div}_{N}}^{eq.assn}+{[{effector}_{div}]}_{T}}-\frac{k_{{die}_{N}}K_{{die}_{N}}^{eq.assn}+k_{{div}_{N}}^{*}\left[ {effector}_{div} \right]_{T}}{K_{{die}_{N}}^{eq.assn}+\left[ {effector}_{div} \right]_{T}}-\frac{k_{N\to Y}K_{N\to Y}^{eq.assn}+{k_{N\to Y}^{*}\left[ {effector}_{tsn} \right]}_{T}}{K_{N\to Y}^{eq.assn}+\left[ {effector}_{tsn} \right]_{T}}-k_{N\to A}-k_{N\to A2} \right){[N]}_{T}+\frac{k_{A\to N}K_{A\to N}^{eq.assn}+{k_{A\to N}^{*}\left[ {effector}_{tsn} \right]}_{T}}{K_{A\to N}^{eq.assn}+\left[ {effector}_{tsn} \right]_{T}}{[A]}_{T}+k_{A2\to N}{[A2]}_{T}+k_{Y\to N}{[Y]}_{T}$$

[*effector_div_*], [*effector_tsn_*]: each can be either [Y] or [A2] + [Y]

: if effects on division and death are present

: if effects on transitions are present

: if the A→N (and A→A2) transitions are present

: if the N→A (and A2→A) transitions are present

: if the N→Y (and A2→Y) transitions are present

: if the Y→N (and Y→A2) transitions are present

: if the N→A2 and A2→N transitions are present

Subtype A2:

$$\frac{dA2}{dt}=\left( \frac{k_{{div}_{A2}}K_{{div}_{A2}}^{eq.assn}+{k_{{div}_{A2}}^{*}[{effector}_{div}]}_{T}}{K_{{div}_{A2}}^{eq.assn}+{[{effector}_{div}]}_{T}}-\frac{k_{{die}_{A2}}K_{{die}_{A2}}^{eq.assn}+{k_{{div}_{A2}}^{*}\left[ {effector}_{div} \right]}_{T}}{K_{{die}_{A2}}^{eq.assn}+\left[ {effector}_{div} \right]_{T}}-\frac{k_{A2\to Y}K_{A2\to Y}^{eq.assn}+k_{A2\to Y}^{*}\left[ {effector}_{tsn} \right]_{T}}{K_{A2\to Y}^{eq.assn}+\left[ {effector}_{tsn} \right]_{T}}-k_{A2\to A}-k_{A2\to N} \right){[A2]}_{T}+\frac{k_{A\to A2}K_{A\to A2}^{eq.assn}+{k_{A\to A2}^{*}\left[ {effector}_{tsn} \right]}_{T}}{K_{A\to A2}^{eq.assn}+\left[ {effector}_{tsn} \right]_{T}}{[A]}_{T}+k_{N\to A2}{[N]}_{T}+k_{Y\to A2}{[Y]}_{T}$$

[*effector_div_*]: in candidate models where A2 is neuroendocrine, effector is [Y]; candidates where A2 is non-neuroendocrine, effector is [A] +[N] (neuroendocrine subtypes decrease non-neuroendocrine growth (3))

[*effector_tsn_*]: can be either [Y] or [A2] + [Y]

: if effects on division and death are present

: if effects on transitions are present

: if the A→A2 (and A→N) transitions are present

: if the A2→Y (and N→Y) transitions are present

: if the Y→A2 (and Y→N) transitions are present

: if the A2→A (and N→A) transitions are present

: if the N→A2 and A2→N transitions are present

Subtype Y:

$$\frac{dY}{dt}=\left( \frac{k_{{div}_{Y}}K_{{div}_{Y}}^{eq.assn}+{k_{{div}_{Y}}^{*}[{effector}_{div}]}_{T}}{K_{{div}_{Y}}^{eq.assn}+{[{effector}_{div}]}_{T}}-\frac{k_{{die}_{Y}}K_{{die}_{Y}}^{eq.assn}+{k_{{div}_{Y}}^{*}\left[ {effector}_{div} \right]}_{T}}{K_{{die}_{Y}}^{eq.assn}+\left[ {effector}_{div} \right]_{T}}-k_{Y\to N}-k_{Y\to A2}-k_{Y\to A} \right){[Y]}_{T}+\frac{k_{N\to Y}K_{N\to Y}^{eq.assn}+{k_{N\to Y}^{*}\left[ {effector}_{tsn} \right]}_{T}}{K_{N\to Y}^{eq.assn}+\left[ {effector}_{tsn} \right]_{T}}\left[ N \right]_{T}+\frac{k_{A2\to Y}K_{A2\to Y}^{eq.assn}+{k_{A2\to Y}^{*}\left[ {effector}_{tsn} \right]}_{T}}{K_{A2\to Y}^{eq.assn}+\left[ {effector}_{tsn} \right]_{T}}{[A2]}_{T}+k_{A\to Y}{[A]}_{T}$$

[*effector_div_*]: [A] +[N] (neuroendocrine subtypes decrease non-neuroendocrine growth (3))

[*effector_tsn_*]: can be either [Y] or [A2] + [Y]

: if effects on division and death are present

: if effects on transitions are present

: if the N→Y and A2→Y transitions are present

: if the Y→N and Y→A2 transitions are present

: if the A→Y transition is present

: if the Y→A transition is present

References

1. Harris LA, Beik S, Ozawa PMM, Jimenez L, Weaver AM. Modeling heterogeneous tumor growth dynamics and cell–cell interactions at single-cell and cell-population resolution. Curr Opin Syst Biol. 2019 Oct 1;17:24–34.

2. Gurung, S, Perocheau, D, Touramanidou, L, Baruteau, J. The exosome journey: from biogenesis to uptake and intracellular signalling. *Cell Communication and Signaling*. 2021. *19*(47). https://doi.org/10.1186/S12964-021-00730-1

3. Mollaoglu G, Guthrie MR, Böhm S, Brägelmann J, Can I, Ballieu PM, et al. MYC Drives Progression of Small Cell Lung Cancer to a Variant Neuroendocrine Subtype with Vulnerability to Aurora Kinase Inhibition. Cancer Cell. 2017 Feb 13;31(2):270–85.
