# Supplementary material for: Unified tumor growth mechanisms from multimodel inference and dataset integration
Source: PLoS Comput Biol. 2023 Jul 5;19(7):e1011215. doi: 10.1371/journal.pcbi.1011215 (PMC10351715; doi:10.1371/journal.pcbi.1011215)
Supplement: S3 Text — (DOCX) [file pcbi.1011215.s003.docx]

**S3 Text.** **Simulations using best-fitted parameters, as opposed to randomly-selected parameters from the prior distributions, replicate subtype proportions at steady state**

While all tumors in each dataset are SCLC tumors, (based on p53/Rb mutations (1) and histological assessment) each dataset represents a different genetic background of SCLC. (In the case of the SCLC-A cell lines, this “genetic background” is an estimation that the tumors are likely genetically similar because they have similar behavior, i.e. similar major subtype proportions.) Due to these three different tumor behaviors/major subtypes present in each of the three datasets, we represent each subtype’s proportion in each dataset with a different Beta distribution (**Fig 3B**; **Fig S4A-C**, labeled as “probabilistic representation”).

Nested sampling was run per dataset, and differentiated between more likely and less likely models for that particular dataset. Given that model selection determines marginal likelihoods by balancing model complexity with goodness of fit, we expected that the subtype proportions at steady state in the highest scoring models would replicate cell subtype proportions in the data. That is, if nested sampling results in a set of well-fitting parameters per dataset, we expect to simulate the model with these parameters and see that the resulting simulated subtype proportions recapitulate those in the data.

By contrast, we expect that simulating models with non-fitted parameters will not recapitulate data subtype proportions. If non-fitted (possibly randomly-selected) parameters replicate subtype proportions in the data, then nested sampling and fitting will have been unnecessary; that, is it will not have provided any information about appropriate parameters or about which candidate models may best match the data. Therefore, we compare simulating models with non-fitted vs. fitted parameters to both evaluate how well nested sampling was able to fit the models but also to determine that we have gained information during the parameter fitting / marginal likelihood calculation process.

To represent a set of non-fitting parameters, we randomly selected parameters from each prior parameter marginal distribution. These parameter sets with which to simulate the model represent independent selections from each prior distribution (prior distributions for all possible parameters are plotted in **Fig S3**). For the fitted parameters, we sampled from the Bayesian model-averaged joint posterior distribution: each individual parameter set, weighted by the posterior probability of the model for which it was fitted. Other studies have noted the need for sampling fitted parameters from the joint posterior distribution rather than independently sampling from marginal posterior distributions per parameter when simulating models using fitted parameters (2).

Simulating the best-fitting models, (those representing the ideal two-subtype and three-subtype topologies per dataset, such as those investigated in **Fig 5B**) using parameters selected only from the prior marginal distributions, the subtype proportions at steady state for each subtype tended to fall at 0 (0% of the simulated tumor) or 1 (100% of the simulated tumor), indicating that parameters chosen at random from the prior distributions do not fit the data (**Fig S4**). However, in selecting parameters from the posterior, fitted, distributions – those representing the highest-scoring parameter sets – the simulations matched the data much more closely. There still remained simulations where the subtypes fell either at 0% or 100% of the simulated tumor, but the subtype steady state fell within the probabilistic representation of the data more of the time (**Fig S4**).

Investigating the integral of the simulated proportion densities within the 95% confidence interval of the probabilistic representation of the data, (**Fig S4A-C** “integral under prior/posterior”) it is clear that for each dataset the model selection / parameter fitting process was unable to bring simulated subtype steady states completely within the 95% confidence interval of the measured subtype proportions, though it has brought it closer than the prior predictive. We would interpret this as the fact that each dataset used for fitting was not able to completely outweigh the subtype proportions resulting from simulations using the prior parameters, which is a known possibility with regard to using Bayes’ Theorem to fit a model’s parameter sets to data (3).

We highlight also that the posterior predictive density for the Y subtype in the RPM dataset is bimodal (**Fig S4**, middle), which we expect is related to the very wide range of the probabilistic representation of Y proportions in this dataset (**Fig 3A; Fig S4B** “probabilistic representation”). We hypothesize that the constraints imposed on the parameters by fitting to *n-1* subtypes (where n is the number of subtypes in the candidate model): with the need for the same parameters to provide a result within the TKO data density and within the N data density, parameters for Y were likely constrained toward the two peaks seen in the plot rather than falling anywhere within the Y data density.

In all, simulations using fitted parameters regardless of dataset resulted in a 30-50% better correspondence to subtype proportions in the data, indicating that the process of model selection and model averaging resulted in models and parameter sets that were able to represent the data at hand to a satisfactory extent, and that the data was able to inform our SCLC multimodel inference question.

References

1. Rudin CM, Brambilla E, Faivre-Finn C, Sage J. Small-cell lung cancer. Nat Rev Dis Primers. 2021 Dec 1;7(1).

2. Eydgahi H, Chen WW, Muhlich JL, Vitkup D, Tsitsiklis JN, Sorger PK. Properties of cell death models calibrated and compared using Bayesian approaches. Mol Syst Biol [Internet]. 2013 Jan 1 [cited 2021 Aug 16];9(1):644. Available from: <https://www.embopress.org/doi/full/10.1038/msb.2012.69>

3. van de Schoot R, Depaoli S, King R, Kramer B, Märtens K, Tadesse MG, et al. Bayesian statistics and modelling. Nature Reviews Methods Primers 2021 1:1 [Internet]. 2021 Jan 14 [cited 2021 Nov 3];1(1):1–26. Available from: https://www.nature.com/articles/s43586-020-00001-2
