# Supplementary material for: Unified tumor growth mechanisms from multimodel inference and dataset integration
Source: PLoS Comput Biol. 2023 Jul 5;19(7):e1011215. doi: 10.1371/journal.pcbi.1011215 (PMC10351715; doi:10.1371/journal.pcbi.1011215)
Supplement: S4 Text — (DOCX) [file pcbi.1011215.s004.docx]

**S4 Text.** **All datasets support alteration of phenotypic transition rates in the presence of N or A2 subtypes**

The highest likelihood model topologies (**Fig 5A**, blue) for the TKO GEMM data, along with the four-subtype topology, are compared in **Fig 5B** (left). Four model variables have significantly different parameter rates across model topologies: (*i*) the A-to-Y transition, (*ii*) the Y-to-A transition, *(iii*) the A-to-A2 transition, and (*iv*) the A2-to-A transition. The A-to-Y transition has a slower rate if A2 is present in the population and the Y-to-A transition has a faster rate if A2 is present. The presence of N along with A2 increases the rate of the A-to-Y transition, though it is still significantly slower than when N and A2 are absent. Only Y affects the A-to-A2 transition, increasing its rate. N does decrease the rate of the A2-to-A transition despite no effect from Y. These observations have mechanistic implications: A2 may represent an intermediate subpopulation in the tumor that is longer-lived, and will only slowly transition to Y. In the topology with A, A2, and Y (**Fig 5A**, structure 2), the A-to-A2 transition takes up more of the flux in the network (**Fig 5B** left and **Fig S6B**, red arrowheads). Additionally, the N-to-Y transition is faster relative to the A2-to-Y transition (**Fig S6B**, red bar), suggesting that N is a shorter-lived intermediate in the A-to-N-to-Y transition. This result aligns with previous experiments (1) where N was identified as a short-lived state in the A-to-N-to-Y transition. We therefore predict that A2 and N are involved in regulating the relative abundance of, and flux between, A and Y in the tumor.

We also compared the highest likelihood model topologies (**Fig 5A**, red) for RPM-fitted models, as well as the four-subtype topology (**Fig 5B**, middle). Five parameters rates are significantly different across model topologies. These are (*i*) the A-to-Y transition, (*ii*) the Y-to-A transition; *(iii*) the A-to-N transition; *(iv)* the N-to-Y transition; and *(v)* the N division rate. The A-to-Y and Y-to-A transitions are decreased and increased by N, respectively; this is very similar to these transitions in the TKO background, which are also decreased and increased by an intermediate, (in the TKO case, A2) despite the experimental data being different. The presence of A2 along with N decreases the rate of the Y-to-A transition, though it is still significantly faster than when N and A2 are absent. The presence of A2 increases the rate of the A-to-N transition, and decreases the rate of the N-to-Y transition (**Fig 5B**, middle). We thus predict that N and A2 are modulating the transition between, and relative abundance of, A and Y. Unlike in the TKO data, when A2 is present the flux through the system spends more time in the N subtype, with more frequent transitions to N and less frequent transitions to Y (**Fig S6B**, teal arrowheads); additionally, the rate of the A2-to-N transition is faster than the N-to-A2 transition (**Fig S6B**, teal bar). We predict that while N may be a shorter-lived intermediate than A2, A2 regulates the flux from A-to-N-to-Y.

Next, we compared the highest likelihood model topologies (**Fig 5A**, green) for the SCLC-A cell line data and the four-subtype topology (**Fig 5B**, middle). Seven model term parameter rates are significantly different across model topologies, five of which recapitulate rate alterations based on the presence or absence of different subtypes in TKO or RPM datasets. The five rate alterations that parallel those in the TKO or RPM datasets are: (*i*) the A-to-A2 transition, increased by Y (seen in parameter rates on the TKO background), (*ii*) the A-to-N transition, increased by A2 (seen in parameter rates on the RPM background), (*iii*) the A-to-Y transition, decreased both by N (RPM) and A2 (TKO), (*iv*) the Y-to-A transition, increased by both N (RPM) and A2 (TKO), and (*v*) the division rate for subtype N, which increases with Y in both the RPM dataset and the SCLC-A cell line dataset. A sixth model term parameter, that of the A2-to-A transition, is affected in both the TKO and this SCLC-A cell line dataset, but on the TKO background it is decreased in the presence of N, while on the SCLC-A background, it is decreased by the presence of Y. The rate alteration unique to the SCLC-A dataset is the N-to-A2 transition, which is less frequent in the four-subtype topology, indicating that the presence of Y decreases this rate.

Overall, we find that the use of BMA enabled us to uncover similar or identical patterns of parameter rate alterations in the presence of particular subtypes across datasets. We highlight the A-to-Y transition, regulated in a similar manner across all datasets; using multiple independent datasets to find the same or similar effects on kinetic parameter rates lends more weight to our predictions about how the N and A2 subtypes may regulate the system flux from A to Y through intermediates.

Reference

1. Ireland AS, Micinski AM, Kastner DW, Guo B, Wait SJ, Spainhower KB, et al. MYC Drives Temporal Evolution of Small Cell Lung Cancer Subtypes by Reprogramming Neuroendocrine Fate. Cancer Cell. 2020;38(1):60-78.e12.
