# Supplementary material for: Unified tumor growth mechanisms from multimodel inference and dataset integration
Source: PLoS Comput Biol. 2023 Jul 5;19(7):e1011215. doi: 10.1371/journal.pcbi.1011215 (PMC10351715; doi:10.1371/journal.pcbi.1011215)
Supplement: S1 Table — (DOCX) [file pcbi.1011215.s005.docx]

| **Table S1. Existing data pertaining to SCLC intratumoral heterogeneity and communication used for rate parameter priors.** |
| --- |
| NCI-H69 cell line (SCLC-A) doubles at 51.1 +/- 3.1 hours, equivalent to 0.469 doublings per day (1). |
| NCI-H82 (SCLC-N) doubles at 25.5 +/- 4.2 hours, equivalent to 0.783 doublings per day (1). |
| NCI-H841 (SCLC-Y) doubles at 31.2 +/- 3.6 hours, equivalent to 0.769 doublings per day (1). |
| DMS53 (SCLC-A2) doubles at ~127 hours, equivalent to 0.1898 doublings per day (2). |
| Average of apoptotic indices for SCLC cell lines is 0.081, used as 0.081 deaths per day (3). |
| Little is known about timing of phenotypic transitions so a very permissive range was used in the model, with values considered uniformly likely from 0.01 transitions per day (1 transition every ~3 months) to 3 transitions per day. We find this to be a reasonably permissive range based on mechanistic modeling of the epithelial-to-mesenchymal transition in breast cancer cells and stem cell differentiation (4,5), as transition rates for SCLC subtypes have not been reported in the literature. In these reports, the EMT transition was fit to between 10 (4) and 20 (5) days. |
| Changes in growth rates specifically due to inter-subtype effects have not been recorded. NE viability (luciferase) and division (EdU incorporation) are increased by NonNE when plated together (6);  NonNE growth decreases in the presence of NE (7). We used a 5% increase or decrease of the baseline value (depending on subtype and interaction) as the parameter prior for each affected rate. |

References

1. Kaur G, Reinhart RA, Monks A, Evans D, Morris J, Polley E, et al. Bromodomain and hedgehog pathway targets in small cell lung cancer. Cancer Lett. 2016 Feb 28;371(2):225–39.

2. Pettengill OS, Sorenson GD, Wurster-Hill DH, Curphey TJ, Noll WW, Gate CC, et al. Isolation and growth characteristics of continuous cell lines from small‐cell carcinoma of the lung. Cancer [Internet]. 1980 [cited 2021 Nov 4];45(5). Available from: https://acsjournals.onlinelibrary.wiley.com/doi/10.1002/1097-0142(19800301)45:5%3C906::AID-CNCR2820450513%3E3.0.CO;2-H

3. Sirzén F, Zhivotovsky B, Nilsson A, Bergh J, Lewensohn R. Higher spontaneous apoptotic index in small cell compared with non-small cell lung carcinoma cell lines; lack of correlation with Bcl-2/Bax. Lung Cancer [Internet]. 1998 Oct 1 [cited 2021 Nov 4];22(1):1–13. Available from: http://www.lungcancerjournal.info/article/S016950029800066X/fulltext

4. Turner C, Kohandel M. Quantitative approaches to cancer stem cells and epithelial–mesenchymal transition. Semin Cancer Biol. 2012 Oct 1;22(5–6):374–8.

5. Lu M, Jolly MK, Levine H, Onuchic JN, Ben-Jacob E. MicroRNA-based regulation of epithelial–hybrid–mesenchymal fate determination. Proceedings of the National Academy of Sciences [Internet]. 2013 Nov 5 [cited 2021 Nov 4];110(45):18144–9. Available from: <https://www.pnas.o36rg/content/110/45/18144>

6. Lim JS, Ibaseta A, Fischer MM, Cancilla B, O’Young G, Cristea S, et al. Intratumoural heterogeneity generated by Notch signalling promotes small-cell lung cancer. Nature. 2017;545(7654):360–4.

7. Mollaoglu G, Guthrie MR, Böhm S, Brägelmann J, Can I, Ballieu PM, et al. MYC Drives Progression of Small Cell Lung Cancer to a Variant Neuroendocrine Subtype with Vulnerability to Aurora Kinase Inhibition. Cancer Cell. 2017 Feb 13;31(2):270–85.
