# Supplementary material for: Unified tumor growth mechanisms from multimodel inference and dataset integration
Source: PLoS Comput Biol. 2023 Jul 5;19(7):e1011215. doi: 10.1371/journal.pcbi.1011215 (PMC10351715; doi:10.1371/journal.pcbi.1011215)
Supplement: S2 Table — (DOCX) [file pcbi.1011215.s006.docx]

| **S2 Table. Model term posterior probabilities after hypothesis exploration, TKO high-probability 3-subtype topology** | | | |
| --- | --- | --- | --- |
| **Model variable** | **Candidate model prior per hypothesis / summed prior** | **Model-averaged posterior probability** | **Odds ratio** |
| A to N transition | N/A | N/A | N/A |
| A to A2 transition | $P\left( M \vert H_{A\to A2} \right)=0.0008$, $P\left( M \vert H_{no} \right)=0.004$ / sum 0.5 vs 0.5 | $P(H_{A\to A2}\vert D)=0.69$ | 2.23 |
| N to Y transition | N/A | N/A | N/A |
| A2 to Y transition | $P\left( M \vert H_{2\to Y} \right)=0.0008$, $P\left( M \vert H_{no} \right)=0.004$ | $P\left( H_{A2\to Y} \vert D \right)=0.53$ | 1.13 |
| A to Y transition | $P\left( M \vert H_{A\to Y} \right)=0.001$, $P\left( M \vert H_{no} \right)=0.00$2 / sum 0.5 vs 0.5 | $P(H_{A\to Y}\vert D)=0.67$ | 2.03 |
| N to A2 transition | N/A | N/A | N/A |
| A2 to N transition | N/A | N/A | N/A |
| N to A transition | N/A | N/A | N/A |
| A2 to A transition | $P\left( M \vert H_{A2\to A} \right)=0.002$, $P\left( M \vert H_{no} \right)=0.001$ / sum 0.5 vs 0.5 | $P(H_{A2\to A}\vert D)=0.72$ | 2.57 |
| Y to N transition | N/A | N/A | N/A |
| Y to A2 transition | $P\left( M \vert H_{Y\to A2} \right)=0.0015$, $P\left( M \vert H_{no} \right)=0.0011$ / sum 0.5 vs 0.5 | $P(H_{Y\to A2}\vert D)=0.6$9 | 2.23 |
| Y to A transition | $P\left( M \vert H_{Y\to A} \right)=0.0018$, $P\left( M \vert H_{no} \right)=0.0010$ / sum 0.5 vs 0.5 | $P(H_{Y\to A}\vert D)=0.82$ | 4.56 |
| Non-NE affects division & death | $P\left( M \vert H_{div\_eff} \right)=0.0011$, $P\left( M \vert H_{no} \right)=0.0015$ / sum 0.5 vs 0.5 | $P(H_{div\_eff}\vert D)=0.44$ | 0.79 |
| Y affects division & death vs A2&Y affect division & death | $P\left( M \vert H_{div\_eff\_Y} \right)=0.0015$, $P\left( M \vert H_{div\_eff\_A2\_Y} \right)=0.0015$, $P\left( M \vert H_{no} \right)=0.0010$ / sum 0.33 vs 0.33 vs 0.33 | $P(H_{div\_eff\_Y}\vert D)=0.\text{28, }P(H_{div\_eff\_A2\_Y}\vert D)=0.33$ | 0.39, 0.50 |
| Non-NE affects early transitions (A-N, A-A2) | $P\left( M \vert H_{early\_eff} \right)=0.0011$, $P\left( M \vert H_{no} \right)=0.0016$ / sum 0.5 vs 0.5 | $P(H_{early\_eff}\vert D)=0.38$ | 0.61 |
| Y affects early transitions (A to N, A to A2) vs A2&Y affect these | $P\left( M \vert H_{early\_eff\_Y} \right)=0.0015$, $P\left( M \vert H_{early\_eff\_A2\_Y} \right)=0.0015$, $P\left( M \vert H_{no} \right)=0.001$0 / sum 0.33 vs 0.33 vs 0.33 | $P(H_{early\_eff\_Y}\vert D)=0.25\text{, }P(H_{early\_eff\_A2\_Y}\vert D)=0.30$ | 0.33, 0.43 |
| Non-NE affects late transitions (N-Y, A2-Y) | $P\left( M \vert H_{late\_eff} \right)=0.0019$, $P\left( M \vert H_{no} \right)=0.0010$ / sum 0.5 vs 0.5 | $P(H_{late\_eff}\vert D)=0.29$ | 0.41 |
| Y affects late transitions (N to Y, A2 to Y) vs A2&Y affect these | $P\left( M \vert H_{late\_eff\_Y} \right)=0.0026$, $P\left( M \vert H_{late\_eff\_A2\_Y} \right)=0.0026$, $P\left( M \vert H_{no} \right)=0.000$6 / sum 0.33 vs 0.33 vs 0.33 | $P(H_{eff\_from\_Y}\vert D)=0.21\text{, }P(H_{eff\_from\_A2}\vert D)=0.24$ | 0.27, 0.32 |
| If Non-NE effect true, comes from Y or A2&Y? | $P\left( M \vert H_{eff\_from\_Y} \right)=0.0015$, $P\left( M \vert H_{eff\_from\_A2\_Y} \right)=0.0015$ / sum 0.5 vs 0.5 | $P(H_{eff\_from\_Y}\vert D)=0.46$, $P(H_{eff\_from\_A2\_Y}\vert D)=0.54$ | 0.85, 1.17 |
