# Supplementary material for: Unified tumor growth mechanisms from multimodel inference and dataset integration
Source: PLoS Comput Biol. 2023 Jul 5;19(7):e1011215. doi: 10.1371/journal.pcbi.1011215 (PMC10351715; doi:10.1371/journal.pcbi.1011215)
Supplement: S3 Table — (DOCX) [file pcbi.1011215.s007.docx]

| **S3 Table. Model term posterior probabilities after hypothesis exploration, RPM high-probability 3-subtype topology** | | | |
| --- | --- | --- | --- |
| **Model variable** | **Candidate model prior per hypothesis / summed prior** | **Model-averaged posterior probability** | **Odds ratio** |
| A to N transition | $P\left( M \vert H_{A\to N} \right)=0.0014$, $P\left( M \vert H_{no} \right)=0.0066$ / sum 0.5 vs 0.5 | $P(H_{A\to N}\vert D)=0.75$ | 3.0 |
| A to A2 transition | N/A | N/A | N/A |
| N to Y transition | $P\left( M \vert H_{N\to Y} \right)=0.0014$, $P\left( M \vert H_{no} \right)=0.0062$ / sum 0.5 vs 0.5 | $P\left( H_{N\to Y} \vert D \right)=0.61$ | 1.56 |
| A2 to Y transition | N/A | N/A | N/A |
| A to Y transition | $P\left( M \vert H_{A\to Y} \right)=0.0017$, $P\left( M \vert H_{no} \right)=0.0038$ / sum 0.5 vs 0.5 | $P(H_{A\to Y}\vert D)=0.73$ | 2.70 |
| N to A2 transition | N/A | N/A | N/A |
| A2 to N transition | N/A | N/A | N/A |
| N to A transition | $P\left( M \vert H_{N\to A} \right)=0.0027$, $P\left( M \vert H_{no} \right)=0.0020$ / sum 0.5 vs 0.5 | $P(H_{N\to A}\vert D)=0.79$ | 3.76 |
| A2 to A transition | N/A | N/A | N/A |
| Y to N transition | $P\left( M \vert H_{Y\to N} \right)=0.002$7, $P\left( M \vert H_{no} \right)=0.002$0 / sum 0.5 vs 0.5 | $P(H_{Y\to N}\vert D)=0.6$9 | 2.23 |
| Y to A2 transition | N/A | N/A | N/A |
| Y to A transition | $P\left( M \vert H_{Y\to A} \right)=0.003$2, $P\left( M \vert H_{no} \right)=0.0018$ / sum 0.5 vs 0.5 | $P(H_{Y\to A}\vert D)=0.8$2 | 4.56 |
| Non-NE affects division & death | $P\left( M \vert H_{div\_eff} \right)=0.0023$, $P\left( M \vert H_{no} \right)=0.0024$ / sum 0.5 vs 0.5 | $P(H_{div\_eff}\vert D)=0.45$ | 0.82 |
| Y affects division & death vs A2&Y affect division & death | N/A (since A2 as part effect not possible, calculations are same as Non-NE affects division, death, above) | N/A | N/A |
| Non-NE affects early transitions (A-N, A-A2) | $P\left( M \vert H_{early\_eff} \right)=0.0022$, $P\left( M \vert H_{no} \right)=0.0024$ / sum 0.5 vs 0.5 | $P(H_{early\_eff}\vert D)=0.46$ | 0.85 |
| Y affects early transitions (A to N, A to A2) vs A2&Y affect these | N/A (since A2 as part effect not possible, calculations are same as Non-NE affects early transitions, above) | N/A | N/A |
| Non-NE affects late transitions (N-Y, A2-Y) | $P\left( M \vert H_{late\_eff} \right)=0.0039$, $P\left( M \vert H_{no} \right)=0.0016$ / sum 0.5 vs 0.5 | $P(H_{late\_eff}\vert D)=0.37$ | 0.59 |
| Y affects late transitions (N to Y, A2 to Y) vs A2&Y affect these | N/A (since A2 as part effect not possible, calculations are same as Non-NE affects late transitions, above) | N/A | N/A |
| If Non-NE effect true, comes from Y or A2&Y? | N/A (A2 as part of the Non-NE effect not possible) | N/A | N/A |
|  |  |  |  |
