# Supplementary material for: Unified tumor growth mechanisms from multimodel inference and dataset integration
Source: PLoS Comput Biol. 2023 Jul 5;19(7):e1011215. doi: 10.1371/journal.pcbi.1011215 (PMC10351715; doi:10.1371/journal.pcbi.1011215)
Supplement: S4 Table — (DOCX) [file pcbi.1011215.s008.docx]

| **S4 Table. Model term posterior probabilities after hypothesis exploration, SCLC-A cell line data high-prob. 3-subtype topology** | | | |
| --- | --- | --- | --- |
| **Model variable** | **Candidate model prior per hypothesis / summed prior** | **Model-averaged posterior probability** | **Odds ratio** |
| A to N transition | $P\left( M \vert H_{A\to N} \right)=0.0029$, $P\left( M \vert H_{no} \right)=0$.0093 / sum 0.5 vs 0.5 | $P\left( H_{A\to N} \vert D \right)=$ 0.63 | 1.70 |
| A to A2 transition | $P\left( M \vert H_{A\to A2} \right)=0.0029$, $P\left( M \vert H_{no} \right)=0.0093$ / sum 0.5 vs 0.5 | $P(H_{A\to A2}\vert D)=0.54$ | 1.17 |
| N to Y transition | N/A | N/A | N/A |
| A2 to Y transition | N/A | N/A | N/A |
| A to Y transition | N/A | N/A | N/A |
| N to A2 transition | $P\left( M \vert H_{N\to A2} \right)=0.0037$, $P\left( M \vert H_{no} \right)=0.0053$ / sum 0.5 vs 0.5 | $P(H_{N\to A2}\vert D)=0.69$ | 2.23 |
| A2 to N transition | $P\left( M \vert H_{A2\to N} \right)=0.003$7, $P\left( M \vert H_{no} \right)=0.0053$ / sum 0.5 vs 0.5 | $P(H_{A2\to N}\vert D)=0.6$9 | 2.23 |
| N to A transition | $P\left( M \vert H_{N\to A} \right)=0.0057$, $P\left( M \vert H_{no} \right)=0.0036$ / sum 0.5 vs 0.5 | $P(H_{N\to A}\vert D)=0.76$ | 3.17 |
| A2 to A transition | $P\left( M \vert H_{A2\to A} \right)=0.0057$, $P\left( M \vert H_{no} \right)=0.0036$ / sum 0.5 vs 0.5 | $P(H_{A2\to A}\vert D)=0.63$ | 1.70 |
| Y to N transition | N/A | N/A | N/A |
| Y to A2 transition | N/A | N/A | N/A |
| Y to A transition | N/A | N/A | N/A |
| Non-NE affects division & death | $P\left( M \vert H_{div\_eff} \right)=0.0044$, $P\left( M \vert H_{no} \right)=0.0044$ / sum 0.5 vs 0.5 | $P(H_{div\_eff}\vert D)=0.16$ | 0.19 |
| Y affects division & death vs A2&Y affect division & death | N/A (since Y effect not possible, calculations are the same as Non-NE affects division & death, above) | N/A | N/A |
| Non-NE affects early transitions (A-N, A-A2) | $P\left( M \vert H_{early\_eff} \right)=0.0045$, $P\left( M \vert H_{no} \right)=0.0042$ / sum 0.5 vs 0.5 | $P(H_{early\_eff}\vert D)=0.17$ | 0.21 |
| Y affects early transitions (A to N, A to A2) vs A2&Y affect these | N/A (since Y effect not possible, calculations are the same as Non-NE affects early transitions, above) | N/A | N/A |
| Non-NE affects late transitions (N-Y, A2-Y) | N/A (Y not in this model so no transitions toward it) | N/A | N/A |
| Y affects late transitions (N to Y, A2 to Y) vs A2&Y affect these | N/A (Y not in this model so no transitions toward it) | N/A | N/A |
| If Non-NE effect is true, comes from Y or A2&Y? | N/A (Y effect not possible) | N/A | N/A |
