# Supplementary figures and images for: Unified tumor growth mechanisms from multimodel inference and dataset integration
Source: PLoS Comput Biol. 2023 Jul 5;19(7):e1011215. doi: 10.1371/journal.pcbi.1011215 (PMC10351715; doi:10.1371/journal.pcbi.1011215)

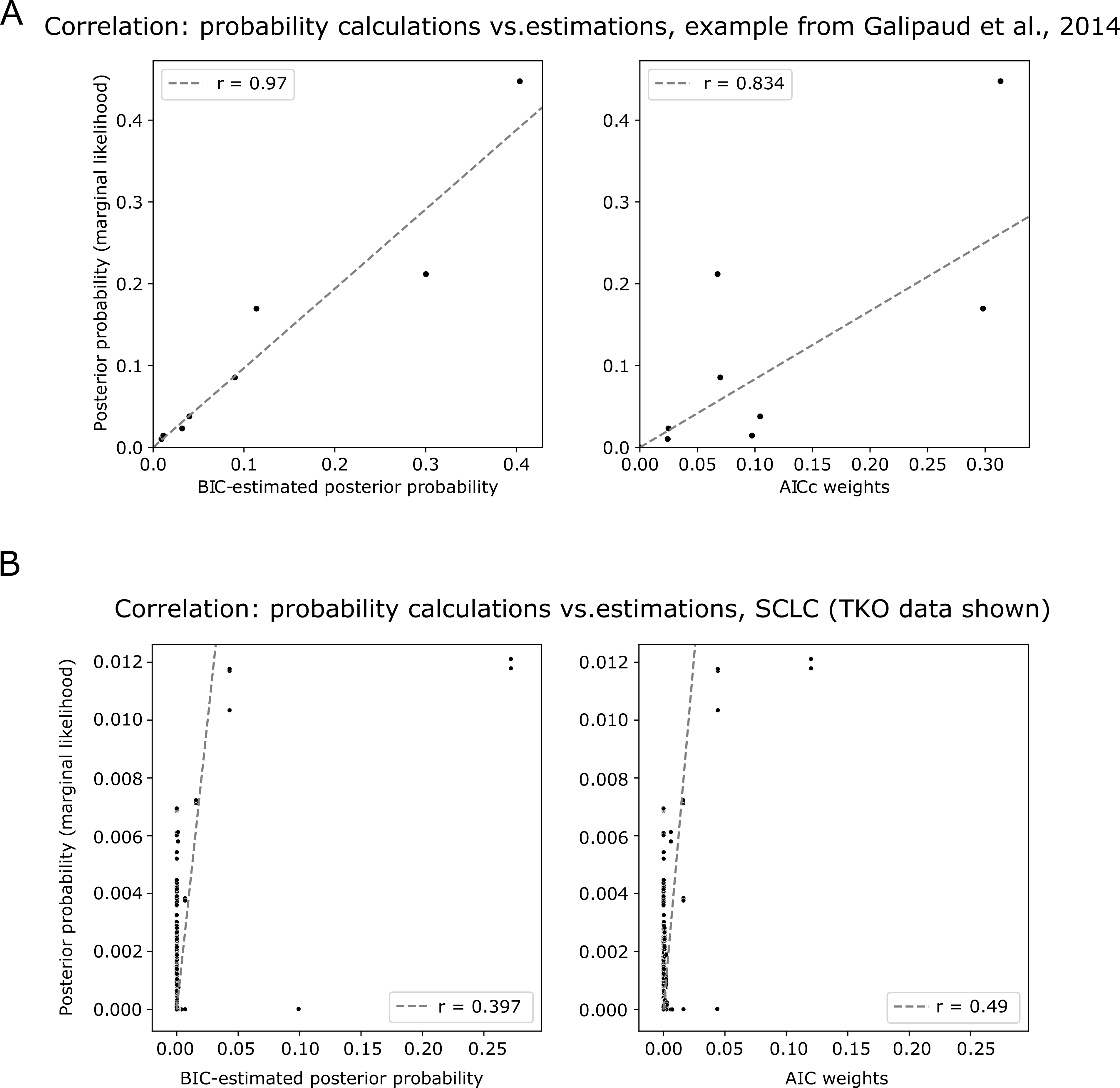

Supplement: S1 Fig — (A) Comparing posterior probability calculations from marginal likelihood returned by nested sampling to posterior probabilities estimated by information criteria, for the 16-candidate model set and simulated data from the linear regression model selection example in Galipaud et al., 2014 [15]. Pearson correlation coefficient (r) is shown for each comparison. Comparison to posterior probability calculated from the BIC-estimated marginal likelihood, left. Comparison to AICc weights, right. (B) Comparing posterior probability calculations from marginal likelihood to posterior probabilities estimated by information criteria, for the SCLC analysis of 5,891 models compared to TKO data. Multimodel inference comparing candidate models to RPM and SCLC-A cell line data provide similar results. Pearson correlation coefficient (r) is shown for each comparison. Comparison to posterior probability calculated from the BIC-estimated marginal likelihood, left. Comparison to AICc weights, right. (TIFF) [file pcbi.1011215.s010.tiff]

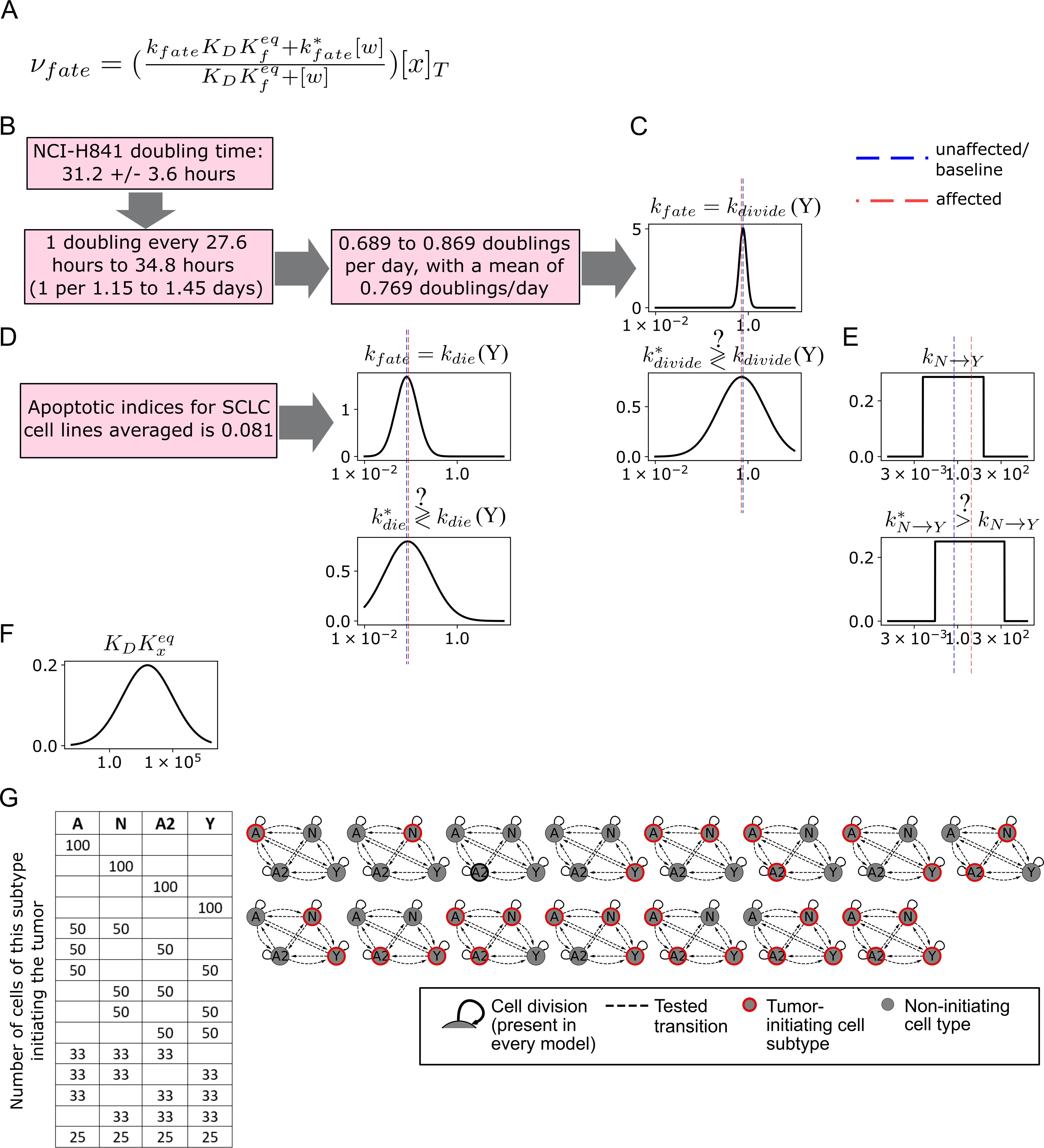

Supplement: S2 Fig — (A) Rate of a cell fate (division, death, or phenotypic transition) for x (vfate) can be calculated as a function of the population size of the effector cell w (see S2 Text Note A) [1]. (B) Example calculation of division rate parameter prior for H841, representation of subtype Y, (see S1 Table) converting doubling times to “per day” units. (C) Division prior for subtype Y, (blue dashed line centered at the mean) as well as inter-subtype effect on division, whose mean is centered 5% lower (red dashed line; see S1 Table) with wider variance to account for more uncertainty in inter-subtype effects. (D) Example calculation and visualization of death rate parameter prior for Y (blue dashed line at mean) and inter-subtype effect on death (red dashed line at mean, 5% higher). (E) Example uniform transition prior, (see S1 Table) here showing N to Y transition; blue dashed line at baseline transition rate center, red dashed line at inter-subtype effect transition rate center. (F) Equilibrium assumption prior, representing KDKxeq in the equation (A). Each affected interaction has a unique KDKxeq prior, but all such priors have identical values (centered at 1000) before fitting. (G) Different model initiation hypotheses, where a model can be initiated by one or more subtypes (thick red outline) depending on the subtypes present in the topology. For an n-subtype topology, there are 2n-1 potential initial conditions. Here, the 4-subtype topology is shown, in a table representing all options for initial number of cells of each subtype (left) and in model schematics (right), with 24−1 = 15 initial conditions. With equal prior probabilities, each hypothesis about which cell types initiate the tumor has a prior probability of 6.67%. (TIFF) [file pcbi.1011215.s011.tiff]

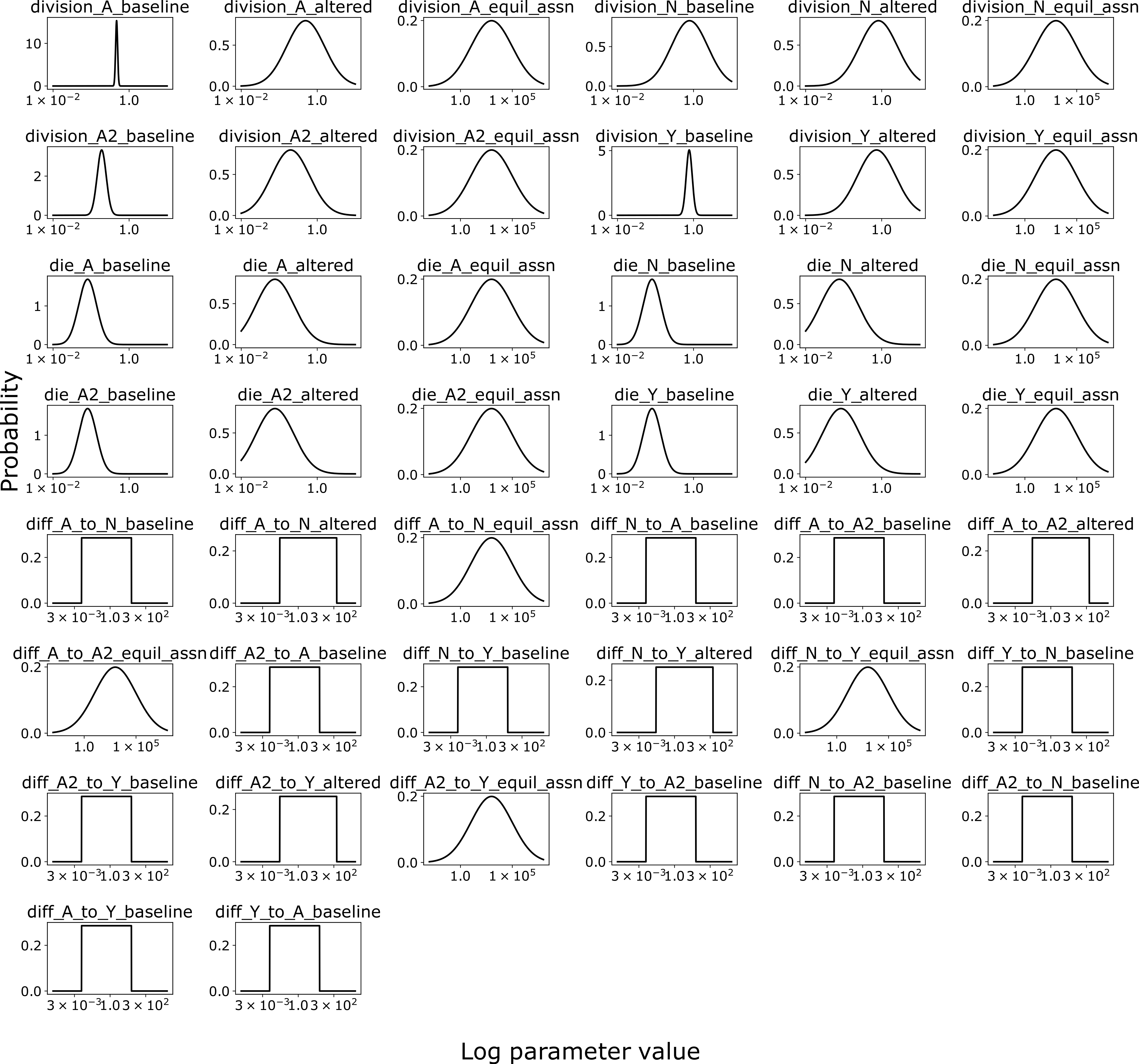

Supplement: S3 Fig — If a candidate model does not contain a reaction, for example a model with the topology A, N, and Y does not include A2 and thus will not include A2 division, death, or transitions to/from A2, then the rate parameter priors for A2-related reactions will not be included as a parameter prior for model fitting. (TIFF) [file pcbi.1011215.s012.tiff]

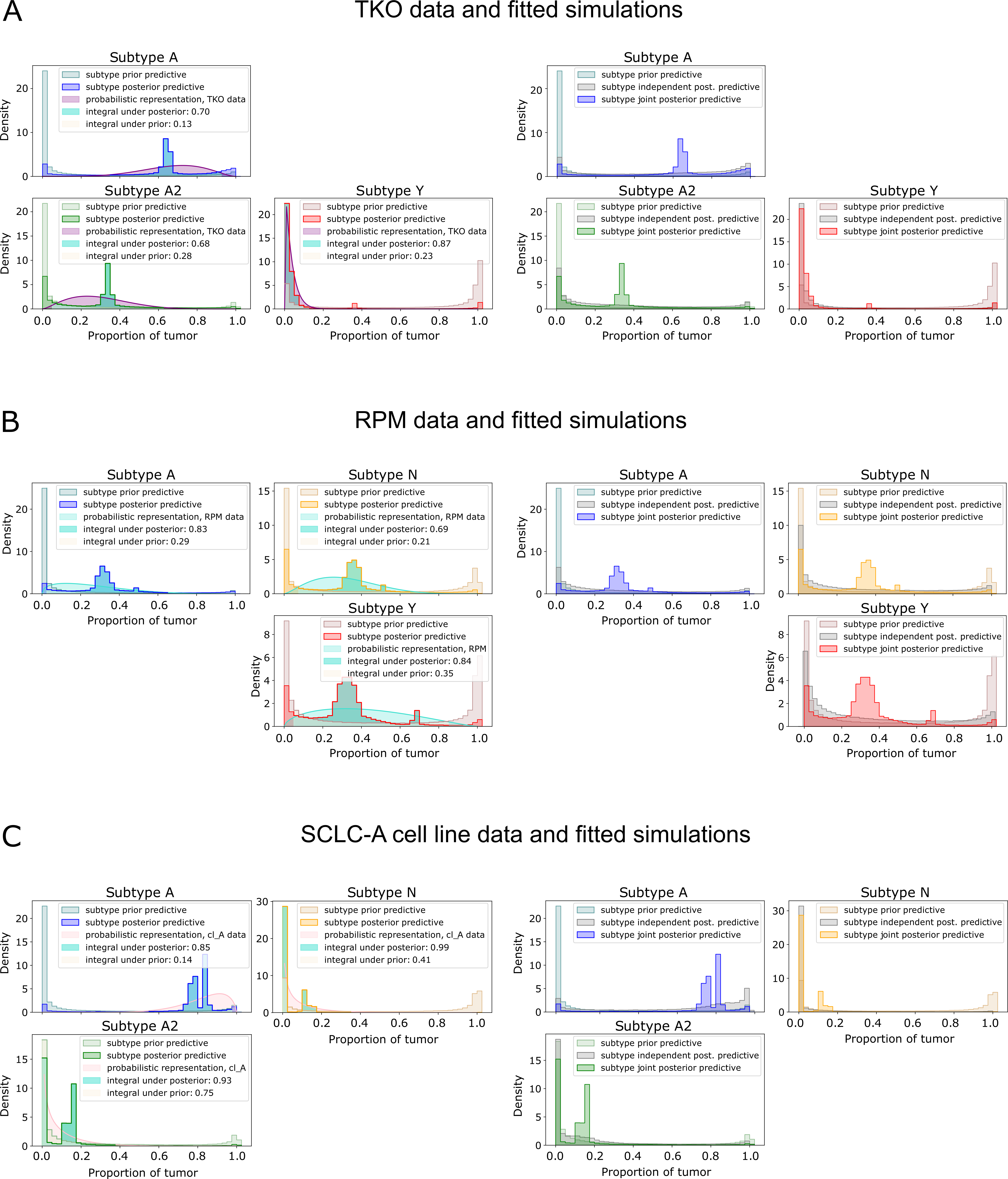

Supplement: S4 Fig — Left, Data distribution, prior predictive distribution, and posterior predictive distribution for each dataset and all candidate models. Data is represented by a Beta distribution, bounded by zero and one, and used in the likelihood function input for Multinest (see Methods). Prior predictive distribution represents model simulations using parameters randomly drawn from the prior. Posterior predictive is generated by model simulations using best-fitting parameters returned by Multinest. Right, prior predictive distribution, simulation steady-state proportions using independently-sampled posterior marginal parameters (“subtype independent post. predictive”), and simulation steady-state proportions using parameters sampled from the joint posterior distribution (“subtype joint posterior predictive”, same as posterior predictive distribution on the left). See S3 Text for more detail and discussion related to these results. Data and predictive distributions for each dataset shown. (A) TKO, (B) RPM, (C) SCLC-A cell lines. (TIFF) [file pcbi.1011215.s013.tiff]

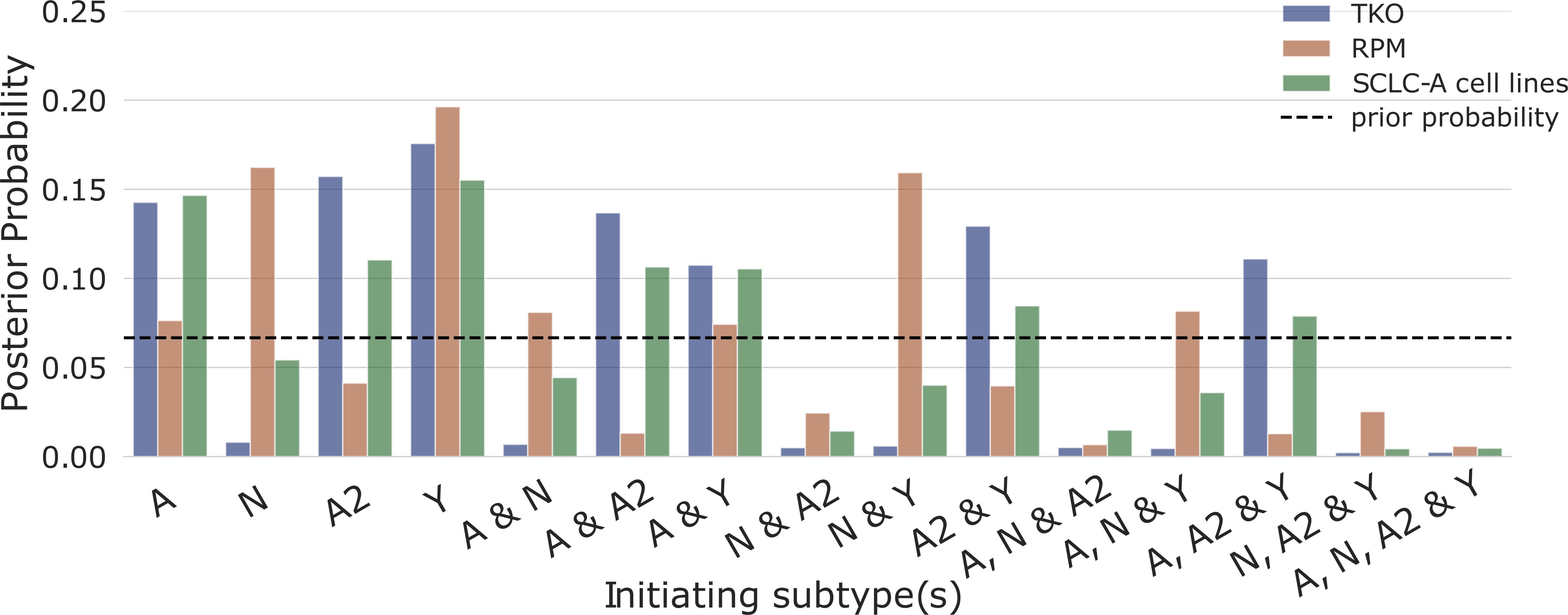

Supplement: S5 Fig — Hypothesis assessment of tumor-iniating subtypes, per dataset. Probability indicates the result of Bayes theorem using equivalent prior probabilities per initiating subtype, black dotted line (located at 6.67% probability that one of the initiation schemes in the x-axis best represents the data) and marginal likelihoods summed per initiation scheme. All topologies (Fig 5A) used in this analysis. (TIFF) [file pcbi.1011215.s014.tiff]

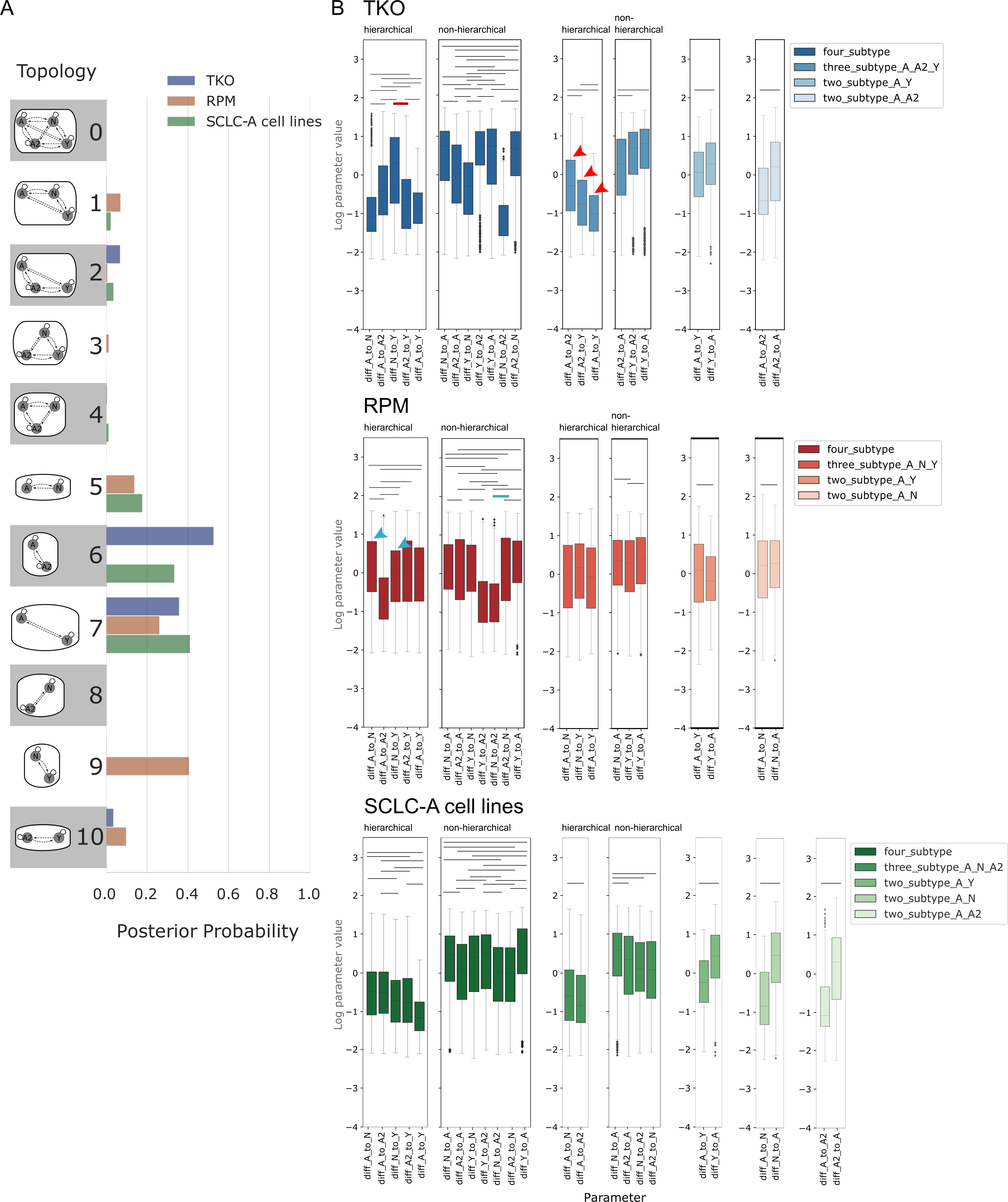

Supplement: S6 Fig — (A) Hypothesis assessment of model topologies per dataset, posterior probabilities based on all candidate models, with no filtering based on initiating subtype (see S4 Fig). Model topologies represented by images and corresponding numbers along the y-axis. (B) Comparison of phenotypic transition parameter posterior marginal distributions, BMA-weighted, per dataset, separated by topology. In 3- and 4-subtype topologies, distributions are further separated by hierarchical or non-hierarchical transition status. Bars indicate significance between samples from BMA parameter distributions at family-wise error rate (FWER) of 0.01, using one-way ANOVA plus Tukey HSD. Red bar: comparing N-to-Y rate with A2-to-Y rate, noted in the main text. Red arrowheads: higher A-to-A2 transition rate in 3-subtype TKO topology (A, A2, Y) compared to A-to-Y and A2-to-Y (noted in Fig 5B as well). Teal bar: comparing A2-to-N rate with N-to-A2 rate, noted in the main text. Teal arrowheads: higher A-to-N transition rate in 4-subtype RPM topology compared to A-to-Y and N-to-Y (noted in Fig 5B). (TIFF) [file pcbi.1011215.s015.tiff]
