# Supplementary material for: Unified tumor growth mechanisms from multimodel inference and dataset integration
Source: PLoS Comput Biol. 2023 Jul 5;19(7):e1011215. doi: 10.1371/journal.pcbi.1011215 (PMC10351715; doi:10.1371/journal.pcbi.1011215)
Supplement: Attachment — Submitted filename: ALTOS Letterhead_2022 CFL PCB.pdf [file pcbi.1011215.s016.pdf]

ALTOS™

Monday, December 19, 22

Dear Editor,

We are excited to submit our manuscript titled *Unified Tumor Growth Mechanisms from Multimodel Inference and Dataset Integration*, for consideration as a research article for publication at PLOS Computational Biology. The formulation of formal mechanisms that describe cellular processes continues to be a fundamental challenge in biology. This problem is exacerbated by the increasing amount and complexity of data generated. Unfortunately, researchers often appeal to informal reasoning or simple correlations to assign mechanistic explanations to systems-level processes, which provides an incomplete understanding of complex systems. This has led to a knowledge space that is mired with multiple (and at times controversial) interpretations of cellular processes even on the same system.

In this work we propose a novel application of multimodel inference and Bayesian statistics that departs from these traditional approaches. We embrace an approach based on multi-model inference and conditional probability to (*i*) find the best model hypotheses to explain a given dataset, and (*ii*) identify how data best informs a mechanistic hypothesis. To demonstrate our approach, we tackle the problem of tumor growth dynamics in small cell lung cancer (SCLC), not only assessing which outstanding hypotheses in the field are supported by data but determining that despite different datasets used in the analysis, similar and unifying conclusions can be drawn about the overall tumor growth mechanisms. We therefore replace informal reasoning and the traditional "one-model" paradigm with a computational hypothesis exploration of thousands of potential explanations where cellular mechanisms are best understood as probabilistic processes – itself a significant paradigm shift.

We have already presented early results of this work at various meetings including the NIH Cancer Systems Biology Consortium annual meetings (2020 (poster award), 2021, 2022 (poster award)), Cold Spring Harbor Labs Cellular Dynamics and Models conference (2021), Systems Approaches to Cancer Biology (2022) and the International Conference on Systems Biology of Human Disease (2022 (poster award)) and found great enthusiasm and interest among our peers. For reviewers, we suggest individuals versed in mechanistic modeling of cellular processes and knowledge about cancer modeling. For example, Dr. Jianhua Xing (U of Pittsburgh), Dr. Sui Huang (Systems Biology Institute), Dr. Steven Wiley (Pacific Northwest National Laboratory), Dr. Fred Adler (U of Utah), or Dr. Eric Deeds (UCLA) would all be well-suited referees to review our work.

We have placed all relevant data files and provided links in Dropbox with anonymous access to reviewers. We will place these files on Zenodo and provide a proper DOI for them once reviewers find them acceptable.

Please let us know if you have further suggestions to help us grow this paper as we strongly believe the message is highly important for all biologists. We thank you in advance for your consideration and time devoted to our work and look forward to your response!

Sincerely,

Carlos F. Lopez, Ph.D.
Principal Scientist, CIPHeR
Lead, Multiscale Modeling Group
