# Supplementary material for: Unified tumor growth mechanisms from multimodel inference and dataset integration
Source: PLoS Comput Biol. 2023 Jul 5;19(7):e1011215. doi: 10.1371/journal.pcbi.1011215 (PMC10351715; doi:10.1371/journal.pcbi.1011215)
Supplement: Attachment — Submitted filename: Response to Reviewers.pdf [file pcbi.1011215.s017.pdf]

Dear Editor,

Enclosed please find a revised and updated version of our manuscript, entitled “Unified Tumor Growth Mechanisms from Multimodel Inference and Dataset Integration”, by Samantha P. Beik *et al.*, to be considered for publication in PLoS Comp Bio. We thank the reviewers for carefully assessing and providing constructive criticisms to our manuscript. In what follows, we present responses to the suggestions and concerns expressed by the reviewers. The reviewer comments (italics) are followed by detailed replies to each comment along with changes made to the manuscript as indicated by each heading. The updated manuscript and supplementary materials contain revisions as needed as well as updated results. We hope this version of the manuscript addresses the concerns raised by the reviewers and look forward to your response.

Sincerely,

Carlos F. Lopez, PhD
(On behalf of all coauthors)

Reviewer #1

Comment 1:

In their manuscript "Unified Tumor Growth Mechanisms from Multimodel Inference and Dataset Integration" Beik et al. introduce a mathematical/computational model selection framework (Bayes-MMI) and apply it to investigate different competing hypotheses about the inner workings of small cell lung cancer comprised of multiple interaction tumor cell populations.
The paper is well written and the methodology of Bayesian Model selection is an adequate approach to compare (and aggregate over) the zoo of thousands of possible mathematical models to describe SCLC dynamics. Moreover appropriate, scalable model selection is an important tool to deal with the ever increasing space of data and hypothesis in current biomedical research. The structure of the manuscript could be improved for the sake of clarity. The main contribution is this new model selection method (Bayes-MMI) and it would deserve its own section in the "Results" (maybe together with some general model selection background). Currently the description of Bayes-MMI (and model selection in general) is interspersed and scattered over several Results-sections discussing mostly the SCLC biology and results.
More importantly, currently the manuscript is missing a few crucial mathematical details, in particular what data was fitted exactly, how the data was fitted, and if the data contains any information to actually distinguish those models.
Any biological conclusions may be highly dependent on those mathematical details.
I hence recommend a major revision to address those shortcomings in the current version of the manuscript. See below for details.

Response: We thank the reviewer for their accurate assessment of our work and recognizing the need for model selection approaches for biological processes. As the reviewer points out, we hope that workflows like Bayes-MMI will become more general for hypotheses exploration and parameter inference. We are also thankful for the reviewer’s candid and constructive feedback. We have done our best to address the concerns raised by the reviewer as described further in the comments below.

Comment 2:

The authors use proportions of cell subtypes (Fig2) as data and their models are fit to that data. From the Methods (l.526) it appears that the authors fit their models' steady state ($X(t=\infty | \theta)$) to this data. How this is done is a bit obscure: The authors calculate "alpha and beta using mean and variance of each (computational?!) dataset" (l.551). The authors evoke a Beta distribution $P(x|\alpha_{sim}, \beta_{sim})$ around their model's (normalized) steady state to calculate the likelihood of an observed datapoint x. $\alpha_{sim}, \beta_{sim}$ are related to the model's normalized steady state, i.e. $\frac{\alpha_i}{\alpha_i+\beta_i}$ = $X_i(\infty|\theta)$. **However it is unclear how the variance of the beta distribution is chosen or why a beta distribution is a reasonable choice.** As ODEs are deterministic, for a given set of parameters θ, the variance in steady state values is zero! The choice of a Beta distribution seems arbitrary. The authors are trying to match to proportions (data and simulation), I don't see an obvious connection to a Beta distribution. If the observed data were

total counts instead of proportions, one could argue for a Dirichlet/Multinomial connection between model and data, with the variance in the data being explained by sampling. This is not the case here.

Response:
We thank the reviewer for pointing out a potential source of misunderstanding in our work. Our choice for likelihood function is due to the kind of data used for our analysis. The available data for this work is the proportion of each SCLC subtype in the tumor type represented by the dataset (i.e. GEMM or composite of cell lines), and we use the mean of each subtype proportion and corresponding variances to generate α and β parameters for a Beta distribution. We selected a Beta distribution as it is a suitable model to capture the behavior of proportions, or relative abundances, as pointed out in previous work by (Paolino, 2001) and (Chai et al., 2018). The Beta distribution is bounded by zero and one, as are our proportions of SCLC subtypes per tumor type, and more naturally captures the relationship between mean and variance that is likely to occur with proportions (Paolino, 2001). In the Beta distribution, a mean close to zero or one generally has a smaller variance than proportions with a mean further from zero or one; in this way, means close to zero and one are likely to have small enough variances for the distribution to be bounded by zero and one without an abrupt cutoff (Paolino, 2001).

For the likelihood calculation of each sampled parameter vector, sampled parameter values are used to simulate the model. We note that n-1 tumor subtype proportions (to ensure independence) from the simulation are taken as mean values for n-1 Beta distributions (one Beta distribution per subtype) and subtype variances from the data are taken as variances for each of the n-1 distributions. For n-1 subtypes, each mean and variance is used to generate their corresponding Beta distributions, at which point the mean proportions for each n-1 subtypes in the data are used as values to calculate the (log) likelihood for a generated Beta distribution. The log likelihoods of each n-1 evaluation of subtype mean at the subtype's generated Beta distribution are then summed (corresponding to the product of the (non-log) likelihoods). We also want to highlight that this is a common way to calculate the log-likelihood $P(D|\theta)$ as noted in (Eydgahi et al., 2013).

Changes to the manuscript:
To address the reviewer's comments, we expanded the previous Methods section and now incorporate a description of our choice for likelihood and Beta distribution in the subsection titled, "Nested sampling marginal likelihood calculation for SCLC candidate models and SCLC datasets."

Comment 3:
As the authors only use steady state data, I don't see how this information is enough to fit those models reliably, let alone do model selection. Similar papers (e.g. [10.1016/j.celrep.2018.11.088](https://doi.org/10.1016/j.celrep.2018.11.088)) use timecourse data with known initial conditions (via cell labeling) and yet still it's hard to distinguish models. Even simple population models (no interaction) will have identifiability issues: Due to the steady

state nature of the data, it's impossible do disentangle growth and death rates. The compositional/proportional nature of the data adds to identifiability issues, hence making model selection tricky. Very different models (with all different kinds of interactions) could give the exact same steady state. Those interactions (e.g. one cell type inhibiting another cell type) would be detectable in correlations (whenever celltype A goes up, celltype B goes down) away from steady state (transient dynamics), but not in steady state. As the framework explicitly assumes independence (the likelihood is a product of beta-distributions), these correlations would be impossible to leverage. Given these concerns and some of the modelling choices (Beta distributions, see 1), it's unclear if the selected models actually explain the data better, or if models cant really be distinguished due to lack of data and model selection just picks the ones that satisfy some of those modeling artifacts.

<u>Response:</u>
We thank the reviewer for this thoughtful question and for alerting us to this similar paper. We find it very interesting that using a similar model selection/model averaging analysis, but with much more specific data (time course data with known initial conditions and cell labeling), is indeed able to inform the investigators' models with more certainty than in our analysis. We agree with the reviewer's overall sentiment that this is likely due to the increased specificity of the data as well as the experimental design with model selection in mind. We further agree that for determining the dynamics of subtype proportion trajectories over time, time course data with known initial conditions and non steady-state conditions would be much better for our SCLC question. Unfortunately, such data is simply not available for SCLC to our knowledge, yet researchers in the field try to infer mechanistic hypotheses from limited available data.

We agree that the dynamics of subtype trajectories between tumor initiation and reaching steady-state are unlikely to be constrained specifically by our multimodel inference process. As such, we choose not to study these pre-steady-state dynamics, but instead focus on model structures constraining any dynamics only to the extent that simulated steady states match steady-state subtype proportions in data (see Figure S4 and Note S6).

We find that, despite the limited data, the model structure (topology, as well as model terms therein) imposes constraints on the possible dynamics of the system. Certainly, topology imposes such constraints, as models without a particular subtype will not provide any trajectory for that subtype. With the constraints we might impose using differing topologies and different combinations of model terms, we wanted to see how many hypotheses of SCLC (previously Table 1, now Table 2) could be informed (either likely to be part of the system that generated the data, or unlikely). Interestingly, the majority of our hypotheses were informed (odds ratio > 2 or < .5; previously Table 2, now Table 3). Those that were not informed are, as the reviewer rightly points out, hypotheses related to cell-cell interactions, which we could not draw conclusions about in this analysis. Even so, we were excited to see that despite the limited data, enough patterns emerge that we can draw conclusions about most of our hypotheses on average.

<u>Changes to the manuscript:</u>

We have added to the Discussion our thoughts related to our ability to learn about SCLC (inform some of our hypotheses) despite the suboptimal data available to us, as stated here. We also have included our considerations related to the fact that without transient dynamics, cell-cell interactions are not identifiable by our analysis. Due to this, we have added that we need more data, especially of the kind in the (Bast et al.) publication the reviewer brought to our attention.

Comment 4:

A general introduction to Bayesian model selection and the Bayes-MMI method deserves it's own Results section independent of it's application to the CCLC data. Maybe a toy example.

Response:

We appreciate the reviewer requesting a more didactic presentation of Bayes-MMI in the Results section. We had in fact already written this result, but it was relegated to the supplement section. To address the reviewer's request, we have moved the section now to the main text.

Changes to the manuscript:

We moved the example portion of Note S1 (was previously Note S2.7) to be the first Results section, as it presents the results using Bayes-MMI with a simple example, comparing Bayes-MMI to AIC, and explaining variable inference across candidate models.

Comment 5:

Most importantly, show the model-class that you're fitting: ODEs. Some mathematical notation might be helpful, also later in the manuscript: $\frac{d X_i}{dt} = f_i(X1,\ldots X_n | \theta)$, X_i being the cell types etc... explain the details of f, growth, death, interaction. I guess it's something like $f_i(X) = \alpha_i + \delta_i X_i + I(X_i, X_j)$. How's that interaction term I modeled? Hill kinetics?

Response:

We thank the reviewer for noting that more detail could be provided related to the ODEs used in our candidate models.

Changes to the manuscript:

We have added more detail to the Methods section discussing the population dynamics modeling in PySB, and a new supplemental note (Note S5) describing the ODEs for each SCLC subtype population in our population dynamics candidate models. Given that this is a model selection analysis, we provide the most complex ODEs per subtype (using all possible parameters) as well as provide notes about when each parameter (and thus each term in the ODE) will be included, based on the candidate model.

Comment 6:

What are the initial conditions of the ODE (are they fitted)? More importantly how's the likelihood function $L(D|\theta)$ defined, and what's the data D in the first place. The authors should be much more explicit about the underlying math!

Response:
We thank the reviewer for requesting additional clarity related to ODE initial conditions, and we provide additional detail in the main text. As noted therein, initial conditions are incorporated into the suite of candidate models: that is, a model with one initial condition is a different candidate than another model with identical rules and parameters but a different initial condition.

Regarding the likelihood function and the data, as we noted in our response to Comment #1, the likelihood function is defined as such: we generate n-1 Beta distributions from alpha and beta parameters calculated using each simulated subtype proportion as the mean and the variance of the data as the variance, then we use each subtype proportion's mean from the data and find the loglikelihood at this value in the corresponding Beta distribution.

Changes to the manuscript:
We added detail in discussing initial conditions, in the Results section "Multiple mechanistic hypotheses emerge from existing data". We also added to Figure S2.G for an additional visual interpretation of initial conditions, including a description in the Figure S2.G legend. Discussion of the likelihood function L(D|theta) and data D has been incorporated in the changes to the text related to Comment #1.

Comment 7:
Clearly separating between new methodology and its application would make the manuscript more concise and avoid the current jumping back and forth between math and biology,

Response:
We thank the reviewer for this suggestion, which we have addressed in moving a previously supplementary Note to the Results (see Response to Comment #4). Now the first Results section of the paper introduces the math and multimodel inference, including explaining variable inference across candidate models. We expect that this will provide the reader with a mathematical overview of our workflow and the methods we use, before moving on to describe our results for SCLC.

Comment 8:
What happens using this approach when faced with a multistable system (not in the data, but on the model side), e.g. two celltypes with self-activation and cross inhibition. Since the authors explore the universe of all (or at least a lot) of model topologies and parameters, one eventually must come across a multistable configuration

Response:

The reviewer raises a very important point. A multistable system would likely have a multimodal parameter/likelihood landscape, where multiple locations in multidimensional parameter space have maxima for log-likelihood values. Fortunately, Multinest, the approach we used for nested sampling, has been shown to capture multimodal landscapes when these are present (first demonstrated in Feroz & Hobson, 2008). Thus, parameter fitting and marginal likelihood calculation are unlikely to be adversely affected by a multistable case.

Comment 9:

Fig4A: node labels are much too small

Response:

Thank you for drawing this to our attention. We have increased the size of the node labels. In the revised manuscript, Figure 4 is now Figure 5.

Comment 10:

The model likelihood is a Beta-distribution around the models mean. How are the individual observations (bars in Fig2) incorporated into the likelihood? Product of likelihoods of each single observation. Or are the observations averaged and only the average proportion (per tumor type) enters the likelihood?

Response:

Each individual observation (bars in what was Fig 2, now Fig 3) represents four subtype proportions in one mouse or cell line sample, summing to 100% (to make up the full tumor sample). Per dataset, the subtype proportions are averaged across samples, so each dataset has four data points, one per SCLC subtype (represented by the colors in the bars in Fig 3), each data point being the mean proportion of that subtype across samples in the dataset, and the corresponding variance in proportion of that subtype across samples in the dataset. As in the answer to comment #1, per dataset, each subtype's mean value is then used as the value for D in P(D|theta) for which the log-likelihood is determined at that value of the corresponding Beta distribution for the subtype. The log-likelihoods per subtype are then summed.

Changes to the manuscript:

We believe that the changes to the manuscript made in response to Comments #1 and #5 have provided the additional detail to the text necessary to answer this question.

Reviewer #2

Comment 1:

I enjoyed reading the main body of the text, and strongly support the approach taken to consider multiple models and use Baysian inference & model comparison – this is exactly how comparing models with data should be done, and the ranking of thousands of models is impressive. However, the novelty of this approach is overclaimed. Reading the introduction, one gets the impression that the authors were the first to apply such an approach, which is clearly not the case.

Response:

We are very grateful for the reviewers' positive comments about our work. We apologize that in our excitement about our work we may have oversold it a bit. Thanks to the constructive comment we have toned down any superlative claims and tried to remain more grounded throughout the paper.

Comment 2:

Rephrase the introduction with respect to novelty about the application of multimodel inference. The field of Bayesian model is well established and you are not the first to apply to this to biological models (and you refer to prior work (ref 44) that already takes the marginal likelihood approach).

Response:

We thank the reviewer for this comment, as it is important to accurately represent the state of the field and our contribution to it. We have edited the introductory text to put forth that Bayesian model selection and model averaging is indeed established and have been previously used across disciplines. However, we do consider our approach to be novel in the connection between biological hypotheses (e.g., statements in Table 2) and mechanistic model equations representing each hypothesis (e.g., ODEs and related calculations in Note S4 and Note S5), with these equations tested via the existing Bayesian methodology and finally recapitulated as probability of data support for the underlying biological hypotheses. We have strived to make our novel contributions clearer in the paper.

Changes to the manuscript:

We have edited the Introduction to specify that these features of Bayesian multimodel inference have been previously used with success.

Comment 3:

Relating to the above, you show your BMMI to outperform AIC. A commonly used alternative to AIC is BIC, which can be derived as an approximation from the marginal likelihood (as can be readily read on Wikipedia). Would the BIC offer a computationally more efficient way to get the same results (as you don't have to integrate the posterior distribution)?

Response:

We thank the reviewer for noting the importance of BIC and a potential comparison with AIC and the marginal likelihood in our paper. We note that in the simple Galipaud et al. example (Ref 12 in the manuscript), we find high comparability between BIC- and marginal-likelihood derived posterior probabilities. Galipaud and colleagues themselves found that BIC resulted in improved variable inclusion probabilities over AIC, as we do, though they did not study the marginal likelihood.

For the SCLC data problem considered in this manuscript, we find poor correspondence between these values: the trend between BIC and posterior probabilities is much worse in our SCLC analysis compared to our analysis of simulated data from Galipaud et al. (and is also poor between AIC and posterior probabilities). We have provided Figure S1 to illustrate this, plotting the correlation between posterior probability calculated via marginal likelihood and posterior probability from BIC-estimated marginal likelihood (exp(-BIC/2)) or AIC weights. Although there is congruency in the BIC and marginal likelihood posterior probability results for the simple Galipaud example, we consider the BIC approach simply overwhelmed by the complexity of the problem when considering the more complex SCLC example, consequently BIC fails to have even reasonable agreement with marginal likelihood. We believe this is due to (*i*) the complexity of the SCLC data parameter space, and (*ii*) the fact that marginal likelihood considers every likelihood calculated in the entire parameter space, while AIC and BIC only use *the best likelihood value* within the parameter space (and ignore the rest of the space). We therefore theorize that AIC and BIC are simply missing details about the parameter space that marginal likelihood within our Bayes-MMI workflow is taking into consideration.

Changes to the manuscript:

We added a supplemental note, Note S3, describing BIC and BIC-derived posterior probabilities, and comparing its calculations to those of AICc weights and posterior probabilities from our Bayes-MMI workflow in the simple Galipaud et al. example. In Note S3 we also compare posterior probabilities or model weights generated by Bayes-MMI marginal likelihood, BIC, and AIC for our SCLC analysis. We have made a corresponding figure, Fig S1, showing how posterior probabilities/model weights correspond reasonably well across methodologies in the Galipaud et al. example, but that the correspondence is poor for the SCLC model hypotheses space. We include in Note S3 some thoughts on the complexity of the parameter space and how this is likely a significant contributor to the lack of correspondence between the BIC and Bayes-MMI workflow.

Comment 4:

How is likelihood computed (given that the model is deterministic)? The SI note refers to “least-squares likelihood function” (implying Gaussian errors?), whereas the methods mention a beta function (motivated by sampling).

Response:

We thank the reviewer for pointing out this important aspect, which was also raised by Reviewer #1, and thus noting the need for us to clarify how likelihood has been calculated in our manuscript. Below we describe our use of Beta function and parameter sampling, but also refer the reader to our response to Reviewer #1, Comment #1.

Model fitting is performed via nested sampling (Multinest/PyMultinest) in both the linear regression “toy” example in Results section “Bayesian inference efficiently infers parameter inclusion in the “true” model” and the SCLC multimodel inference problem in the main manuscript. As these data are different, the likelihood functions for model fitting and marginal likelihood calculation are different, though in both cases fitting is performed by Multinest/PyMultinest (nested sampling).

For the linear regression example in “Bayesian inference efficiently infers parameter inclusion in the “true” model”, the “data” (simulated “ground truth” data) comprises multiple arrays, one per variable (five total: one response, $\vec{y}_{data}$, and four predictors, $\vec{x}_1$, $\vec{x}_2$, $\vec{x}_3$, and $\vec{x}_4$, where the arrow indicates an array rather than a scalar). Sampling, for example for the four-variable candidate model, is carried out throughout the five-dimensional parameter space (four coefficients and the intercept) and the predictor variable array values are multiplied by the corresponding coefficient β_i (one sampled scalar value per coefficient). That is, the likelihood function calculates $\vec{y}_{sim}$,

$$\vec{y}_{sim} = \beta_0 + \beta_1\vec{x}_1 + \beta_2\vec{x}_2 + \beta_3\vec{x}_3 + \beta_4\vec{x}_4 \tag{1}$$

and then uses the result to compare this simulated response variable array to the data array,

$$\frac{(\vec{y}_{data} - \vec{y}_{sim})^2}{\sigma^2_{\vec{y}_{data}}} \tag{2}$$

where using the least-squares objective function to compare the simulated response variable array to the “data” array is equivalent to the log-likelihood, assuming normal errors. Candidate models with fewer predictors undergo this same process except coefficients β_i for variables x_i that are not included in the candidate model are not sampled, and set to zero for the likelihood function.

For the SCLC analysis, the data is the mean proportion of each SCLC subtype in the tumor type represented by the dataset (either a GEMM or composite of cell lines). Sampling occurs across the 44-dimensional parameter space, depending on which parameters are present in the candidate model. The sampled parameter values are used for model simulation and the resulting n-1 subtype proportions (n-1 to ensure independence) are taken as mean values and data variances taken as the variances to generate a Beta distribution per subtype, from which the (log) likelihood of the corresponding subtype’s mean in the data is calculated. This is the typical way to calculate the (log)likelihood P(D|θ) (see e.g. Eydgahi et al., 2013).

<u>Changes to the manuscript:</u>
We reorganized the section “Parameter estimation and evidence calculation by nested sampling”, in the Methods section, into three sections. The first (still titled “Parameter estimation

and evidence calculation by nested sampling") now introduces parameter estimation, the general likelihood and posterior probability equations, and nested sampling. The second section, "Nested sampling marginal likelihood calculation for comparison to AIC analysis," which is a new section we added to address the reviewer's concerns and describes in greater detail the data used in the example and sampling across parameter space to perform parameter fitting in a linear regression model. We also added details to Note S1.7, including a snippet of data, to increase the clarity of the methodology in this example for the interested reader. The third section, "Nested sampling marginal likelihood calculation for SCLC candidate models and SCLC datasets," describes in greater detail the data used for model fitting, and augments the description of the likelihood function present in the initially-submitted manuscript.

<u>**Comment 5:**</u>

Why not try and run inference on all 3 datasets together? How would this compare to the consolidated model in Fig 5e? It's not obvious to me that they would be the same, and the former seems the more principled approach.

<u>Response:</u>

We thank the reviewer for this insightful question. Each of the SCLC datasets used for our analyses comes from a specific genetic background in the case of each GEMM, or has a similar composition and thus we group them together, inferring that similar composition is based on similar genetics. We consider the different genetics (or estimates of likely genetics) to result in different tumor behaviors and different major subtypes present in the tumor, despite all being SCLC tumors (both based on p53/Rb mutations and histological assessment). The differences in behavior and major subtype composition can be noted in what was Figure 2, now Figure 3, where samples from the TKO dataset look different from samples in the RPM dataset. Based on our likelihood function, evaluating a sampled parameter value by comparing the resulting simulations to each dataset is equivalent to averaging subtype compositions across the three datasets and then fitting to those proportions. Using all data points, averaging across the three datasets results in an averaged subtype composition that looks like the TKO dataset, in which case information that could be learned from the RPM or SCLC-A cell line datasets would be lost. Even sampling from the TKO dataset, such that all datasets contribute the same number of datapoints to the overall average, results in a misleading subtype composition that is not representative of SCLC biology. In this case no information is gained about any known SCLC system.

We therefore decided to use all three of these datasets, despite not representing identical tumor behavior, because we wanted to learn about SCLC tumor growth mechanisms as a general problem rather than for one specific SCLC context. We interpret each dataset to comprise a snapshot of a different aspect of SCLC as a disease, and therefore were interested in combining each snapshot into one unifying view.

<u>Changes to the manuscript:</u>

We have added text throughout the manuscript more thoroughly describing our views that the differing genetics (or estimated genetics) across datasets describe different possible behaviors of the SCLC tumors. We also describe our choice to consolidate the datasets into one potential unifying model, and further describe our choice not to run inference on all three datasets together as explained here, in the section "Model analysis supports a non-hierarchical differentiation scheme among SCLC subtypes."

Comment 6:

Can you show joint posterior distributions for (some of) the top models, to show whether some parameters are correlated/only identifiable in combination?

Response:

We expect that many, if not all, parameters are correlated upon model fitting. We can see this by simulating a model post-fitting and comparing the simulated SCLC subtype proportions to the subtype proportions seen in our data. As in (Eydgahi et al., 2013) sampling parameter values independently from the posterior marginal distributions of each parameter results in a poor fit of simulated subtype proportions to data, while taking parameters from the joint posterior distribution - using best-fitting parameter sets as returned by Multinest - results in simulated subtype proportions that match the data much more closely. This indicates to us that the values of many parameters are dependent on the values of others.

Changes to the manuscript:

Previously, Fig S4 denoted independent sampling from the parameter prior marginal distributions compared to the joint posterior distribution, but we have added the comparison between independently-sampled posterior parameters and parameter sets from the joint posterior. We have also edited Note S6, which corresponds to Fig S4, to better explain these results.

Comment 7:

Could you make some concrete predictions of experiments that would be most informative to further distinguish between top ranked models, or to be most informative of your consensus model? E.g. by simulating from the model and determining where the predictions deviate most.

Response:

We thank the reviewer for raising this point, as based on our predictions of highly likely SCLC tumor features (hypotheses that received high probability in Table 3) we have considered which future experiments may be helpful in validating these predictions. We had considered the Y-to-A transition prediction to be valuable to assess, as this was the highest probability model term / biological hypothesis across our models and datasets. Labeling SCLC-Y cells, sorting them to a pure population, and measuring whether SCLC-A cells appear, would validate our prediction that SCLC-Y cells can indeed undergo a phenotypic transition to become SCLC-A phenotype cells. In fact, soon after our initial submission of our manuscript, a paper was published where experimentalists evaluated this (and other) phenotypic transitions by fluorescence labeling: with

regard to the Y-to-A transition, they tracked individual SCLC-Y cells, recording that the fluorescent label changed from that for Y to that for SCLC-A, indicating a phenotypic transition within the SCLC cell. They ascertained other phenotypic transitions as well, all in an untreated context. Thus, their publication (Gopal et al., 2022) could serve as validation for several of our predictions: further, given that we used small sets of steady-state data, the recent publication by Gopal and colleagues demonstrates that with limited data, our method could be used to guide further experiments.

<u>Changes to the manuscript:</u>
We have added text to the Discussion describing this concrete suggestion for an experiment to validate our Y-to-A prediction as well as discussing that this previously unmeasured transition has now been published by another group. We also added our thoughts about the importance of their overall findings, both for our predictions of nonhierarchical phenotypic transitions / plasticity in general, as well as for future work comparing treated and untreated samples to evaluate what role plasticity plays in treatment resistance.

Minor points:
A. In line 78 you state you are interested in the case where hypotheses cannot be exhaustively enumerated, then in line 86 you state that you enumerate hypotheses…

<u>Response:</u>
We thank the reviewer for bringing this to our attention. We consider our enumeration of possible models *not* to be "exhaustive": with 44 parameters and 15 initial conditions possible with which to build a model, exhaustively searching the model space would represent $15*2^{44}$ models, and we are searching a vastly smaller number than that.

<u>Changes to the manuscript:</u>
We have added some detail to the main text, in section "Multiple mechanistic hypotheses emerge from existing data", discussing how we consider that we are searching biologically relevant hypothesis space, but are not exhaustively searching model space. We provide more exact numbers to indicate the small subset of models we investigate.

B. Good visual summary of existing hypotheses in Fig 1, however Fig1 B-E is somewhat confusing to follow.

<u>Response and changes to the manuscript:</u>
We thank the reviewer for this feedback. Now Fig 1 is Fig 2, and we have simplified Fig 2 B-E and shifted explanatory text to the figure legend.

C. Fig S2 caption refers to Box 1, and in the bioRxiv preprint I can see boxes, but not in the version provided to me by the journal. (If the boxes are to be used: In Box 1C, is it a realistic assumption that signalling factor f is free after binding to x (rather than remaining bound to x)?*

Response:

We thank the reviewer for highlighting this mistake. We have rewritten the Fig S2 caption to refer to Note S4 rather than Box 1.

With regard to the assumption that signaling factor `f` is free after binding to `x`, we consider signaling factor `f` to represent an exosome or other secreted factor. It has been shown that exosomes generate signaling in recipient cells through direct binding *or* uptake, with direct binding potentially leaving the exosome outside the cell and thus free to interact with other cells once signaling in the initial recipient is initiated; it has also been proposed that exosomes may undergo multiple cell uptake and release cycles, allowing signaling affecting multiple cells (Gurung et al., 2021). Given the ability to continue inducing signaling in multiple cells, we do not consider factor `f` to be removed from the environment upon initiating the signal that activates `x` into `x*`.

Changes to manuscript:

We have rewritten the Fig S2 caption to refer to Note S4 rather than Box 1 (original Note S1, now Note S4, in this manuscript originally had the same content as Box 1 in the bioRxiv, though now they are not identical due to edits to Note S4). We have explicitly included text on the quasi-steady-state and quasi-equilibrium assumptions used in developing our SCLC model with cell-cell interactions in Note S4.

D. Is it valid to use BMA to average posterior parameter distributions? (can you provide a reference?)

Response:

According to a review by Fragoso and colleagues, BMA is fairly commonly used to average posterior parameter distributions. In their review, the authors provide the mathematical basis for combining parameters from multiple models, weighted by posterior probability, leading to combined parameter estimates or predictions (Fragoso et al. 2018). Evaluating 820 publications across multiple disciplines including ecology, statistics, and systems biology, they determined that nearly one third of the publications studied used model averaging to combine fitted parameter values from all models to then simulate with the averaged parameters to generate a combined prediction; nearly one quarter of the publications studied used BMA to estimate a parameter common across models using fitted parameter values and model weights.

Changes to the manuscript:

The review from Fragoso and colleagues is cited in the initially-submitted manuscript, but we have edited the text in section “All datasets support alteration of phenotypic transition rates in the presence of N or A2 subtypes” to include a citation when discussing using BMA to average across posterior parameter distributions.

E. Fig 4B is also hard to read, as Fig 5f

Response and changes to the manuscript:

Thank you for pointing this out. Figures 4 and 5 are now figures 5 and 6 in our revised manuscript, and we have enlarged these figures as well as the text within them.

Reviewer #3

The manuscript addresses a stubborn challenge in computational systems biology and does so with an inventive and rigorous methodology that could have substantial impact to the field. They demonstrate it's applicability using a challenging and medically important question with SCLC. Interpretation of highly complex analysis is clear throughout and the biological implications clearly highlighted. The broad applicability of the method is challenged by the computational complexity of the approach, and it is demonstrated on a single biological question, but this is expected given the size of the challenge the authors are trying to overcome. Given increasing access to increasing computing power the methodology this is not likely to be a barrier to future utility in diverse biological areas.

Addressing the concerns below will ensure the broad applicability of the approach is clear to readers.

Comment 1:

Can the authors clarify the paragraph starting on line 176, and whether each candidate model was chosen due to the potential to capture all the data in table 1. My concern is that some data in table 1 is based on a single reference, and one on unpublished correspondence. As such the reliability of each observation could be challenged by future studies. I would suggest including candidate models that explain all but 1 entry in table 1, where the non-included entry is either a single study or unpublished.

Obviously the time for running the workflow may preclude this analysis but the authors should address how incorrect data in table 1 would affect subsequent results. Any analysis that sheds some light on how each property described in Table 1 impacts the number of candidate models and subsequent results would be powerful in understanding how the literature impacts the outcome of the approach.

Response:

We thank the reviewer for pointing out a potential source of confusion from the data in Table 1, which now in the revised manuscript is Table 2. Each bullet point in the table represents a hypothesis or theory of SCLC tumor behavior. The candidate mathematical models were generated based on the mathematical interpretation of each of these behaviors. However, for hypothesis exploration, each bullet point in the table is considered on its own as well as in combination with the other bullet points. It is all combinations of these hypotheses that give rise to the 5,891 candidate models explored in this work. As the reviewer accurately noted, some candidate models indeed include hypotheses/theories based on *all* data in the table, but some candidate models include partial information, or only a single bullet point. This enables us to compare each of these hypotheses to the data as shown in Table 3, where probabilities and odds ratios are presented for each hypothesis from Table 2, and in what was previously Figure 5 but is now Figure 6.

Changes to manuscript:

We have added clarifying text in the section “Multiple mechanistic hypotheses emerge from existing data,” to discuss how each of the hypotheses in Table 1 will be incorporated or excluded from candidate models during model evaluation.

Comment 2:

Can the authors explain the shape of the probability distribution in Figure S3. The “digital “ distribution such as diff_A_to_N_Baseline? These distributions suggest some parameters values are impossible at a specific value and possible for another one arbitrarily close.

Response:

In Figure S3, these “digital” distributions are uniform prior marginal distributions for model parameters. This is a standard way to select prior parameters in a less-biased manner when one does not have reason to prefer certain prior values over others (Bolstad & Curran, 2017). Because rates of (non-EMT) phenotypic transitions between subtypes in SCLC, or in any other tumor as far as we are aware, have been measured, we chose not to use a normal distribution that would place more weight on certain rates. We used published mathematical model rates of EMT, as this is one type of phenotypic transition, to guide the priors for SCLC phenotypic transitions (see Table S7). We then expanded the range of rates found in those publications to a more permissive range, which we then modeled with uniform priors so as to give equal weight across the range of potential values.

Changes to the manuscript:

We added text to in Results section “Multiple mechanistic hypotheses emerge from existing data” to refer interested readers to Table S7.

Comment 3:

Similarly the shape of the density distributions in Fig S4 should be explained. Subtype Y shows a bi/tri-modal distribution with areas of low density, based on the RPM data. Please describe how these arise. Does the SCLC-A cell line data and TKO data suggest the modes between [0.2,0.4] and [0.6,0.8] are not real as they are not recapitulated by different data sources?

Response:

In Fig S4, we show Beta distribution representations of the data; we maintain this to be the underlying distribution of the data as the measurements and data points are proportions (please also see changes to Methods section explaining the use of Beta distributions). Due to the different tumor behaviors and major subtypes represented, each subtype in each dataset is represented by a different Beta distribution. We decided to use all three datasets, despite not representing identical tumor behavior, as they each represent a snapshot of a different aspect of SCLC as a disease, thus contributing different aspects of overall SCLC tumor behavior. We consider each dataset as a part of the whole SCLC, and thus aim to use all three of these together to learn about SCLC growth mechanisms as a general problem, rather than a problem

in a specific context / in a specific genetic background (see Response to Reviewer #2's Comment #5).

In Fig S4, we aim to indicate that while resulting subtype proportions from simulations using parameter values represented from prior marginal distributions do not overlap with the data distributions (i.e., sampled prior parameters result in a poor fit of model to data), after optimization with Bayes-MMI, the joint posterior parameter distribution enables simulation results of subtype proportions to overlap more closely with the data. We quantify this by calculating the integral under the prior predictive distribution and under the posterior predictive distribution within the subtype proportion values (x-values in Fig S4) that represent between 5% and 95% of the probability density of the data. We expect, and indeed find, that the integral under the posterior predictive distribution measured within the data distribution is closer to 1 than the integral under the prior. This indicates that the distribution of subtype proportions has moved much closer to the data after parameter fitting - thereby indicating that parameter fitting informed the model.

<u>Changes to manuscript:</u>
We have rewritten Note S6, which refers to Figure S4, to improve clarity about the sources of the data probabilistic representation and the prior and posterior predictive distributions (simulated subtype proportions upon using prior marginal or joint posterior parameter distributions). We added text to Note S5 as well discussing our considering of a genetic background or inferred genetic background.

<u>Comment 4:</u>
While I know the analysis is not aiming to find a single best model, and small models have an "unfair" advantage, figure 4 suggest compelling evidence that topology 7 has some underlying truth to it, as the only topology that has very high probability in all datasets. Despite this the authors focus on topologies 1,2, and 4, arguing that 2-node topologies such as topology 7 do not capture all the subtypes required. Can the authors expand on why topology 7 was not taken forward?

<u>Response:</u>
We thank the reviewer for noting this unintended omission. In our approach, we had to strike a balance between empirical features from SCLC experiments, and features amplified by our multimodel inference analysis. We know from the SCLC literature that more than two subtypes have been observed in SCLC tumors. In fact, from the experimental data used for fitting (Figure 3 in our revised manuscript), it appears that at least *three* cell subtypes are present in each data source. Mathematically, the model selection process tends to prioritize smaller models. Therefore, given that two subtypes tend to dominate the experimental datasets, it is not surprising that the two-subtype topology yields the most evidence. However, the question we are trying to answer is not "what is the most likely model supported by the data?" but rather, "what information does the data yield for cell-cell interactions in tumor growth models of up to four subtypes?"

Changes to the manuscript:
We have added text in section “Multiple mechanistic hypotheses emerge from existing data,” discussing why three- or four-subtype models are more relevant for our analysis, yet included two-subtype models for less bias in our workflow. We also provide additional text about moving forward with three-subtype topologies in the section “High-likelihood model topologies are nonoverlapping between datasets”.

Comment 5:
Why does their modelling not shed doubt on the experimental evidence that there is complex multi-subtype tumor composition, as a simple model fits the data best? Can the authors discuss why their approach might identify an unsuitable topology as a “winner” and how this might be avoided when others apply their technique? How could one identify topologies that might not align with experimental evidence if they have high likelihood in multiple datasets? How could the approach be adjusted to ensure an overly small model doesn’t “win”?

Response:
We thank the reviewer for raising a very interesting question. Without any prior knowledge about tumor composition and heterogeneity, we agree that selecting the best scoring model is the best practice. However, given prior knowledge about tumor composition from cell biology, we were able to further constrain our exploration of the hypotheses. Overall, the answer to this question relies largely on the kind of data available for multimodel inference, and we hope our results will motivate experimentalists to collect data that better informs mechanisms, such as time-course data. For this work we only had tumor steady-state data as well as non-quantitative data about heterogeneous tumor composition. However, we point out that having done the calculations for simpler models enabled us to perform the comparisons shown in Figure 5B and draw conclusions about trends in model-averaged parameter changes upon introducing additional subtype(s) to the model; this analysis would not have been possible otherwise.

To increase the likelihood of finding a “winning” model that is not overly simplified, using more, and more specific, data, would be our suggested approach. A more comprehensive likelihood function that could test the simulation’s correspondence to data both with and without treatment would enable more efficient use of data. A more rich experimental trajectories dataset would require the models to incorporate multiple observed subtypes and enable improved fits for more complex models.

Changes to the manuscript:
We have added edits throughout the Discussion section to clearly state both our interpretation of the model selection results, including the higher likelihood of the two-subtype topology, and our approach to hypothesis exploration in this specific context. We have provided more detail about our incorporation of less-quantitative prior knowledge into our analysis and thus why we choose to use the three- or four-subtype topology(ies) for our posterior probability and variable inclusion analyses.

Comment 6:

I do not understand how a "consolidated three-subtype topologies", which produces a "unifying 4 subtype topology" (Figure 5E), can be a reliable unifying mechanism when the 4 subtype topology did poorly in Figure 4A (topology 1)?

Response:

We thank the reviewer for this question, and find that the answer is similar to that for comment #3, above. We agree that the four-subtype topology does not yield the best fit in our multimodel inference analysis, but this was expected given the available datasets. The available data provides only partial information about tumor composition subtypes. However, our prior knowledge from prior work as well as reports in the literature, indicate that the existence of four subtypes in the same tumor is very likely.

We consider the four- subtype topology noted in Figure 5 in the initial submission, now Figure 6, to be a potential unifying mechanism as it enables us to explain the behavior of the three different tumor types (TKO GEMMs, RPM GEMMs, and SCLC-A cell lines) via one model. However, we agree that it is important to consider the context of the question one is working to answer; to predict TKO-specific behavior, we could certainly choose to make predictions using the three-subtype model in revised Figure 6B. We do not aim to suggest that the four-subtype model is the "truth", but that if our prior expectation of the accessibility of four subtypes is accurate, (which, as addressed above, we cannot truly test with this data) we can evaluate the probability of cell subtype behaviors based on the data we have.

Changes to the manuscript:

We have added our thoughts related to this question into the Discussion of the manuscript, including discussing the context of desired predictions that could be made in the future with our model(s). We highlight in the Discussion that our main goal is to evaluate how likely each hypothesis in revised Table 2 is based on the data we have, which in the majority of cases was informative (either "likely" or "unlikely"), but could also be noninformative (an SCLC hypothesis not answerable based on our data, such as cell-cell interactions).

Minor Comments:

1. *While "recalcitrance" is a term used in the SCLC field, but given the broad applicability of the methods described perhaps a term more broadly understood outside the cancer field would be appropriate? "treatment resistant" or just "poor survival"? At least for the abstract.*

Response & change to manuscript:

We thank the reviewer for raising this point, and have substituted "treatment resistance" for "recalcitrance" in the abstract.

2. *I would disagree that Akaike Information Criterion, (AIC) has had limited success as it has been used in countless papers to select the most appropriate model. Can the authors point to any examples where AIC failed to select the right model?*

Response:
We thank the reviewer for pointing this out, and agree that AIC has indeed been generally successful in selecting the most appropriate model. However, we do contend, and hope to have demonstrated in our "toy example" and discussions of sums of AIC weights in SCLC compared to posterior probabilities in SCLC (Figure S1), that AIC is more limited in its success with regard to determining "parameter importance" or the probability for inclusion of a variable in the model.

Changes to the manuscript:
We have updated text in the Introduction, discussion of AIC vs Bayesian MMI in the new Results section about the Galipaud et al. example, and the reasoning behind our choice to use the marginal likelihood for our SCLC analysis in section "Bayesian exploration of candidate population dynamics models using experimental data." In each location we have aimed to bring across AIC's success in model selection but potential limitations in model averaging / variable inclusion.

3. *The introduction suggests that Bayes-MMI is most applicable when "models cannot be exhaustively enumerated", but then to demonstrates the approach by exhaustively enumerating: "mechanistic hypotheses" to generate "thousands of candidate population dynamics models". This suggests their test case may not be the best example. I would suggest rewording this text as it sets the wrong expectation.*

Response:
We thank the reviewer for bringing this to our attention. As stated in response to Reviewer #2 comment A, we consider our enumeration of possible models not to be "exhaustive", and have added some detail to the main text to this effect. With 44 parameters and 15 initial conditions possible with which to build a model, exhaustively searching the model space would represent $15*2^{44}$ models, and we are searching a significantly smaller number.

Changes to the manuscript:
As noted in our response to Reviewer #2 comment A, we have edited the section "Multiple mechanistic hypotheses emerge from existing data", discussing how we consider that we are not exhaustively searching the model space in that we include only a very small percentage of all the possible candidate models we could have generated with 44 parameters and 15 initial conditions.

4. *NE is used (in the intro) before it is defined (in the results)*

Response & change to the manuscript:
We thank the reviewer for pointing this out and have used “neuroendocrine” in place of “NE” in the intro before later defining it in the results.

5. *TFs in Figure 1 are too small to see.*

Response & change to the manuscript:
We thank the reviewer for bringing this to our attention and have increased the size of TFs, and for the smallest TFs we have replaced names with letters that we then specify in the Figure legend. Figure 1 is now Figure 2 in the revised manuscript.

6. *Figure 1 B suggests that C(4,1)=1, surely this should be 4?*

Response & change to the manuscript:
We thank the reviewer for noting our mistake, and have corrected it to C(4,4) = 1 (choosing all 4 subtypes for the model with 4 subtype possibilities results in only one possible topology).

7. *It is not clear how the media is conditioned in the bullet point in table 1, or what the exosomes are isolated from? Is it from a mixed SCLC culture or a particular subtype?*

Response & change to the manuscript:
We thank the reviewer for the opportunity to add more detail related to the conditioned media / exosomes, and have specified in what was Table 1, but is Table 2 in the revised manuscript, that the conditioned media and purified exosomes are from a cell culture of Hes1+ (Non-NE) cells.

8. *I do not agree with this sentence: “Therefore, our results indicate that the data used for model fitting has informed our knowledge about the system, because before nested sampling, all models are equally likely” The authors chose to make all models equally likely, which is correct methodologically, but they likely had intuition based on evidence that some were more likely than others. Almost any approach could move from equal likelihood to something non-equal so I would suggest either removing or rewording this claim.*

Response to comment:
We chose all models to be equally likely *a priori* to prevent bias, even though we know that some are more likely than others (and as the reviewer notes, this is a common methodology). While indeed any approach could move from equal to non-equal likelihood, we consider that the data has informed our knowledge in considering the candidate models in the aggregate.

Whatever the change from equality to non-equality might be, we expect that if the data did not inform the models (imagine if the data were poorly related to the models) the marginal likelihood results would be similar (though non-equal) in value, as no model could be fit well enough to the data to receive a significantly different marginal likelihood. With similar but non-equal marginal likelihood results, in the aggregate, approximately 95% of the models would fall into the 95% confidence interval - approximately 95% of the models would take up 95% of the probability space. However, in our analysis, after model fitting and marginal likelihood calculation, 14-26% of the models account for 95% of the probability space / fall into the 95% confidence interval. In this way we consider that we have gained information from the data within the model hypothesis space.

<u>Changes to the manuscript:</u>
We have specified these details in the text and reworded the claim that the data used for model fitting has informed our knowledge, in the section "A small subset of candidate tumor growth models is supported by experimental data."

9. *The authors state that "we found that the fitted parameter distribution outcome was dependent on choice of initiating subtype Fig S5" I actually find it remarkable how independent of initiating subtype almost all distributions are. I believe the need to narrow down based on initiating subtype can be justified without this extra step. Perhaps it should have been included in number 1.*

<u>Response:</u>
We thank the reviewer for this comment and have explained in the text that we aimed to narrow down by initiating subtype, *but*, given that each dataset was unable to inform an initiating subtype (i.e. posterior initiating subtype probabilities were similar to prior initiating subtype probabilities) we chose to narrow down based on hypotheses about SCLC initiation in the SCLC field: namely, that NE subtypes, and likely A, initiate a tumor.

<u>Changes to the manuscript:</u>
We have edited the text in section "High-likelihood model topologies are nonoverlapping between datasets," so as not to claim that initiating subtype choice affects parameter distributions. We discussed also that we still aimed to narrow down models to average over based on initiating subtype, and could not do so using the data as initiating subtype hypotheses were not informed by the data. To this point, we have changed Fig S5 to show the small difference between prior and posterior initiating subtype probabilities rather than posterior marginal parameter distributions by initiating subtype.

10. *Please make the letter's in the model schematics clearer in figure 4A as the two node topologies all look identical and are only discriminated by tiny black on grey letters that are not legible when printed.*

<u>Response & changes to manuscript:</u>

Thank you for bringing this to our attention. We have increased the size of the node labels. Figure 4 in the initial manuscript is now Figure 5 in the revised manuscript.

11. *Rather than Fig 4B left, third-darkest blue can the authors indicate the important point with a red arrow.*

Response & changes to manuscript:
Thank you for the helpful suggestion. Figure 4 from the initial manuscript is now Figure 5 in the revised manuscript; we have indicated important points with arrowheads in revised Figure 5B and indicated these same comparisons in Fig S6B.

12. *Some of the references appear as internet sources when they are in fact published in journals.*

Response & changes to manuscript:
Thank you for alerting us to this. We have carefully revised our references for accuracy.
