# Supplementary material for: Unified tumor growth mechanisms from multimodel inference and dataset integration
Source: PLoS Comput Biol. 2023 Jul 5;19(7):e1011215. doi: 10.1371/journal.pcbi.1011215 (PMC10351715; doi:10.1371/journal.pcbi.1011215)
Supplement: Attachment — Submitted filename: Response to reviewers 5.17.23.pdf [file pcbi.1011215.s018.pdf]

Dear Editor,

Enclosed please find a revised and updated version of our manuscript, entitled "Unified Tumor Growth Mechanisms from Multimodel Inference and Dataset Integration", by Samantha P. Beik *et al.*, to be considered for publication in PLoS Comp Bio. We thank the reviewers for re-assessing the manuscript after edits from the initial set of reviews, which we believe improved the manuscript greatly. In the following document, we present responses to the question and suggestion from reviewer #1. The reviewer comments (italics) are followed by detailed replies to the comment, then changes made to the manuscript as indicated by the heading. The updated manuscript contains these revisions. We hope this version of the manuscript addresses the concerns raised and look forward to your response.

Sincerely,

Carlos F. Lopez, PhD

(On behalf of all coauthors)

Reviewer #1

<u>Comment 1:</u>

The revised version of their manuscript "Unified Tumor Growth Mechanisms from Multimodel Inference and Dataset Integration" addresses all the points I raised with the original manuscript. The modeling itself is introduced more explicitly and kept separate from the biological system under investigation. The structure of the manuscript is now much clearer. Thank you, I appreciate the effort!

The authors agree with my statement about the limited information content in their steady state data and mention that in the discussion, stating that cell-cell interactions are "uninformed by the data".

I think this might be an issue beyond cell-cell interactions (i.e. the abundance of a cell type influencing division/death/transition of another). Even cell-cell transitions could be unidentifiable. For example, take the model depicted in Fig.4C as ground truth (two cell types x and w, both dividing and dying, plus a unidirectional transition from x to w). Given just deterministic steady state data of this model, I don't see how one would be able to distinguish a model with a non-zero transition rate k_{x->w} from a model where both cell types are completely separated from each other (k_{x->w}== 0) unless the division and death rates are known/fixed. One would not be able to tell which topology is the correct one given the steady state data.

I would like the authors to briefly comment if this identifiabilty issue indeed extends to the transitions rates. Since the transition rates between the different cancer-cell subtypes are a major focus in the later part of the manuscript, this should to be clarified.

<u>Response:</u>

We thank the reviewer for this follow-up question. We agree as previously that the dynamics of subtype trajectories between tumor initiation and reaching steady-state are unlikely to be constrained specifically by our multimodel inference process, and as the reviewer points out, we also agree that any fitted parameter values are likely not well constrained by the limited data at hand. However, our goal was not to predict parameter rates in this paper but rather to compare trends among competing hypotheses given the data. Given the changes in prior *vs.* posterior probabilities we can deduce that for some rates the data does indeed inform the parameter. We present the parameters to compare and identify trends across model topologies in a qualitative rather than quantitative manner.

At the same time, it is well established that a given model topology and its accompanying dataset will limit the possible parameter values, in isolation or in combination, to a degree. For example, work by Sethna (Gutenkunst et al. 2007) showed that despite parameter sloppiness and parameter unidentifiability, it is possible to infer dynamic processes from a given dataset. Indeed, a chosen model topology constrains the system to a subset of possible dynamic modes. Therefore the hypotheses tested and emergent general trends from parameter fitting inform about possible system dynamics. Given that the majority of our hypotheses were informed by the data (odds ratio > 2 or < .5; Tables 2 and 3), we conclude that this analysis provides biologically relevant information about the underlying SCLC tumor growth mechanisms. While we are not able to determine *the* biological mechanism conclusively, we show that the data are more likely to support the cellular transition hypotheses over the single-cell of origin hypotheses, and thus we provide a way forward to test these ideas with further experiments.

<u>Changes to the manuscript:</u>

To further address the reviewer's concerns, we have added a sentence to the Introduction concretely defining the scope of this work. We also edited the introductory section and added a paragraph to the section, "All datasets support alteration of phenotypic transition rates in the presence of N or A2 subtypes," specifying that we aim to compare trends in model-averaged parameter rate distributions across model topologies rather than predict exactly what these rates may be in varying model topologies. Finally, we added text to the Discussion specifying that in the scope of our project, we are not aiming to pinpoint phenotypic transition rates, but to evaluate trends, and that we consider that we have achieved our goal of evaluating SCLC hypotheses, most especially the hypothesis of whether phenotypic transitions are more likely than no phenotypic transitions.

<u>Comment 2:</u>

Minor adjustments:

- Fig4B: minus instead of plus signs, as death and transition decrease the amount of x

<u>Response & change to manuscript:</u>

Thank you for bringing this to our attention. We have edited Fig 4B to now have minus instead of plus signs.
